# Decoding the dynamic perception of risk and speed using naturalistic stimuli: A multivariate, whole‐brain analysis

**DOI:** 10.1002/hbm.26652

**Published:** 2024-03-15

**Authors:** Uijong Ju, Christian Wallraven

**Affiliations:** ^1^ Department of Information Display Kyung Hee University Seoul South Korea; ^2^ Department of Brain and Cognitive Engineering Korea University South Korea; ^3^ Department of Artificial Intelligence Korea University South Korea

**Keywords:** dynamic correlation analysis, risk, searchlight analysis, sliding window, speed

## Abstract

Time‐resolved decoding of speed and risk perception in car driving is important for understanding the perceptual processes related to driving safety. In this study, we used an fMRI‐compatible trackball with naturalistic stimuli to record dynamic ratings of perceived risk and speed and investigated the degree to which different brain regions were able to decode these. We presented participants with first‐person perspective videos of cars racing on the same course. These videos varied in terms of subjectively perceived speed and risk profiles, as determined during a behavioral pilot. During the fMRI experiment, participants used the trackball to dynamically rate subjective risk in a first and speed in a second session and assessed overall risk and speed after watching each video. A standard multivariate correlation analysis based on these ratings revealed sparse decodability in visual areas only for the risk ratings. In contrast, the dynamic rating‐based correlation analysis uncovered frontal, visual, and temporal region activation for subjective risk and dorsal visual stream and temporal region activation for subjectively perceived speed. Interestingly, further analyses showed that the brain regions for decoding risk changed over time, whereas those for decoding speed remained constant. Overall, our results demonstrate the advantages of time‐resolved decoding to help our understanding of the dynamic networks associated with decoding risk and speed perception in realistic driving scenarios.

## Graphical abstract

Here, we used naturalistic stimuli to investigated which different brain regions were able to decode dynamic risk and speed. The dynamic rating‐based correlation analysis uncovered frontal, visual, and temporal region activation for subjective risk and dorsal visual stream and temporal region activation for subjectively perceived speed.

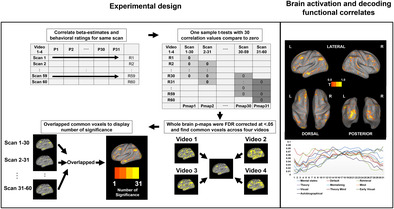

## 1 INTRODUCTION

In the real world, the human brain instantaneously perceives and updates information from its surroundings. To understand how it perceives reality, neuroimaging research must, therefore, investigate how the brain decodes dynamic perceptual changes. However, most neuroimaging studies so far have focused on neural correlates of static perceptual and cognitive processes, using post‐task responses or questionnaires to identify brain regions associated with subjective sensory (Lee Masson et al., 2016), as well as social (Cacioppo et al., 2014) and emotional perception (Putkinen et al., 2020) that do not consider rating as a function of time.

One method to trace dynamic changes in perception is to manipulate stimulus features over time and examine the neural activity associated with these feature changes. This has been done, for example, to investigate stimulus rotation (Chong et al., 2016), image occlusion (Erlikhman & Caplovitz, 2017), dynamic and static face perception (Pitcher et al., 2014), and musical features (Toiviainen et al., 2014). This method is ideal for highly controlled stimuli, but less well suited for investigating perception in real‐world contexts where high‐dimensional changes in stimulus properties occur.

Analyzing verbal reports offers an alternative approach to capturing dynamic perceptions. Using the “think aloud” method (van Someren et al., 1994), changes can be tracked through continuous verbal responses. A number of fMRI studies have used such protocols to decode speech (Moses et al., 2019), game experiences (Klasen et al., 2008), clinical reasoning (Durning et al., 2012), recall (Gilmore et al., 2021), and spontaneous internal thoughts (Van Calster et al., 2017). Although this method has been applied to complex, natural stimuli, one critical limitation is that it can only trace verbalized information actively processed in working memory (Jääskeläinen, 2010). Additionally, it may also slow down general thought processes (Ericsson & Simon, 1984), given the added load and processing cost.

At the same time, studies have mostly used artificial stimuli, as these offer a high degree of control and allow careful manipulation of experimental conditions. This approach, however, comes at the cost of reduced realism and ecological validity (Reggente et al., 2018); accordingly, neuroimaging has recently seen increased use of more natural stimuli, tasks, and viewing environments, employing movies (Yang et al., 2020), games (Ju & Wallraven, 2019), and virtual environments (Chen et al., 2020; Gharib et al., 2020; Kim, Jin, et al., 2020). In the present study, we likewise used naturalistic stimuli to induce dynamic perceptions close to real‐world experiences, building on prior reports that it is possible to use such stimuli even for the decoding of higher level processes such as game experiences (Ju & Wallraven, 2019; Kätsyri et al., 2013; Mathiak et al., 2013), emotion (Antony et al., 2021; Finn & Bandettini, 2021; Kim, Weber, et al., 2020), and auditory attention (Wang et al., 2017). While several previous studies used naturalistic stimuli to decode dynamic perception or cognitive functions like emotional experiences, they did not assess continuous rating while inside the scanner or measure continuous ratings from independent samples (Chan et al., 2020) or the same participants while outside the scanner (Raz, Shpigelman, et al., 2016; Raz, Touroutoglou, et al., 2016), or use physiological signals, such as heart rate, for replace continuous rating (Young et al., 2017). Hence, they were not able to accurately measure subjective experience changes in real time. To address this problem, in the present study we used naturalistic stimuli and implement internal continuous ratings from participants to decode dynamic perceptions that continuously change over time, which has not been accomplished before in previous studies. We hypothesized that continuous ratings would be correlated with neural activity more so than post‐task ratings.

In order to assess dynamic perception that changes over time, we focused on the decoding of the dynamics of the subjective speed of a car and the perceived riskiness of driving: two related factors with different levels of perceptual, emotional, and cognitive processing. Concerning the former, previous studies have exclusively focused on highly controlled stimulus environments, studying aspects of velocity in speed in the context of general motion perception. Brain areas that have been implicated in visual motion perception include the superior temporal sulcus (Krekelberg et al., 2003; Saygin, 2007; Zacks et al., 2006), the middle temporal visual area (Kaderali et al., 2015; Takemura et al., 2012), and premotor areas (Saygin, 2007; van Kemenade et al., 2012). Regarding risk perception in driving, prior studies investigating nonhazardous and hazardous driving have implicated the lateral occipital and right prefrontal cortex (Hirth et al., 2007); these regions are generally associated with attention, emotional processing, stimulus–response, and risk aversion (Megías et al., 2015; Megías et al., 2018). Additionally, an investigation of hazard perception differences between novice and experienced drivers found activation in visual attention networks and occipital and salience network connectivity (Gharib et al., 2020). Prior studies on either speed or risk perception have not examined the dynamics of these processes, which was the purpose of the present study.

To decode dynamic perceptions from neural activity, we asked participants to annotate videos taken on the same race track, but with different drivers, through a joystick‐based interface. For the analysis of brain areas associated with dynamic perceptions, we adopted a sliding window‐based correlation analysis similar to that used in previous studies on the dynamics of functional connectivity (Allen et al., 2012; Hutchison et al., 2013; Mokhtari et al., 2019). Finally, we evaluated the validity of this analysis through an additional “conventional” multi‐voxel pattern analysis based on post‐experiment annotations (similar to Ju & Wallraven, 2019; Kim et al., 2015).

Taken together, through the video‐based driving experiments described in the following, we set out to test four hypotheses: (1) We expected that conventional multi‐voxel pattern analysis would detect brain regions associated with overall risk and speed perceptions. (2) We predicted that brain regions involved in the decoding of dynamic perception would overlap with observations from a conventional searchlight analysis. (3) We expected that continuous rating would decode neural activity better than post‐task ratings (4) Lastly, we hypothesized that brain regions capable of decoding subjective percepts might vary over time.

## 2 MATERIALS AND METHODS

### 2.1 Stimuli

We selected seven YouTube videos recorded from the driver's perspective as stimulus candidates for the fMRI study.

Video 1: https://www.youtube.com/watch?v=7BwDs3ja-WA;

Video 2: https://www.youtube.com/watch?v=F3IHb8RFV_g;

Video 3: https://www.youtube.com/watch?v=mj7dkeQcNPM;

Video 4: https://www.youtube.com/watch?v=QYV3yWshHLI;

Video 5: https://www.youtube.com/watch?v=JvqnUQpa2W4;

Video 6: https://www.youtube.com/watch?v=kqzK9PEgVLA;

Video 7: https://www.youtube.com/watch?v=PlsjIj5O70w&t=314s.

**FIGURE 1:**
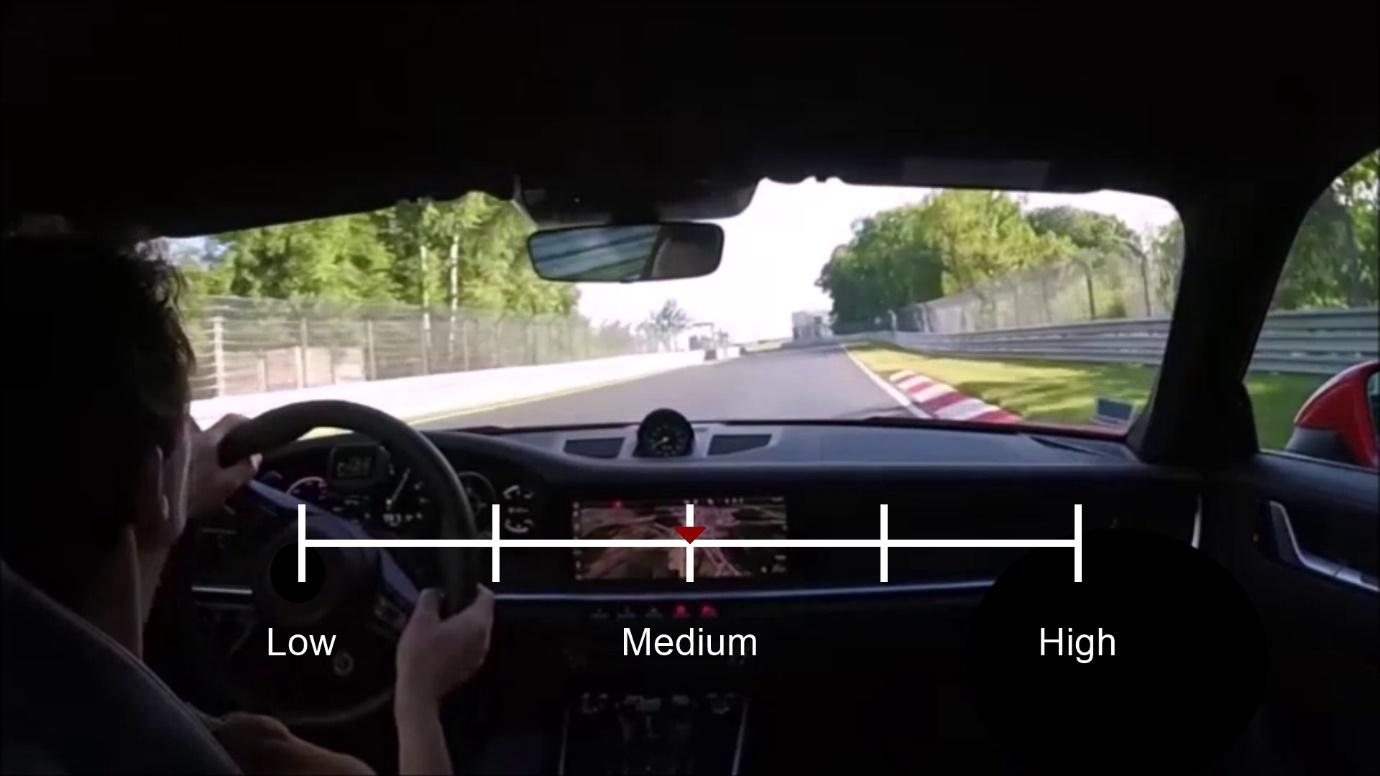
Screenshot of the experiment. A joystick/trackball is used to dynamically rate risk and speed by moving a slider. The red arrow represents the current rating. “Low”, “Medium”, and “High” are assigned to positions 1, 3, and 5 on a Likert scale.

As stimulus candidates for the fMRI study. These videos, all depicting a first‐person view of a car racing on the Nürburgring race track, were uploaded by different users. To make the videos more similar in terms of scene content, they were trimmed to the first 2 min, which included the first part of the lap where the driver departed from the starting line. Additionally, we used Adobe After Effects (https://www.adobe.com/products/aftereffects.html) to hide manufacturer logos and the speedometer with a tracked black mask to obscure information about the type of car and the current speed. Finally, to implement dynamic risk and speed measurements, we used PsychoPy 3.0 (https://www.psychopy.org/) to display a sliding bar on the video clips, which viewers could control with a joystick (see Figure 1).

### 2.2 Experimental design (fMRI experiment)

Before the fMRI experiment, we had a different group of participants evaluate the seven videos to identify those that would allow us to present participants with combinations of high and low risk and high and low speeds, respectively, to cover the maximum range of percepts (see Supporting information, Figure S1, and Table S1 for the methodological details and results). Perceived risk was defined (and explained to our participants) as a subjective assessment of risk, especially in relation to the potential for hazardous situations (Ram & Chand, 2016), while perceived speed was defined as a subjective assessment of the estimated driving speed of the first‐person vehicle (similar to Hussain et al., 2019). Based on the results of our evaluation experiment, we selected four of the seven videos for the fMRI study.

Next, to enable dynamic ratings inside the fMRI scanner, we converted the dynamic rating measurements from the joystick to an fMRI‐compatible trackball (Current Designs, Philadelphia, United States), by correlating the horizontal movement of the trackball with the horizontal movement of the slider. The videos were projected on a PROPixx screen (VPixx Technologies, Quebec, Canada) with a width of 58 cm and a height of 30 cm (1920 × 1080 pixels at 500 Hz), visible to participants from inside the scanner. During the experiment, participants used the trackball to dynamically rate subjective risk in the first block and subjective speed in the second block. After each video, they rated average risk (in block 1) or speed (in block 2) for an overall assessment. Dynamic ratings were recorded for each frame and then averaged across videos to construct a 4 × 4 behavioral matrix for the correlational searchlight analysis. These ratings were averaged every 2 s for the dynamic correlation analysis.

### 2.3 Participants

Thirty‐one right‐handed participants were recruited via university community sites (21 male; mean age, 22.5 years (SD = 3.07)). All the participants were Sungkyunkwan University students who had possessed a driver's license for more than 1 year at the time of the experiment (mean, 3.4 years, SD = 2.50) and reported driving at least once in a year (mean 1574 km/h, SD = 3077.80). No participant had a history of neurophysiological diseases or any other conditions that prevented them from participating in the MRI experiment. The study was approved by the local ethics committee of Korea University (KUIRB‐2020‐0207‐02), and informed consent was provided by all participants, after the experimental procedure had been explained to them and before they underwent the fMRI scanning procedure.

### 2.4 Procedure

Participants received information about the fMRI experiment prior to participating and watched a 30‐s example video taken from the evaluation that was not part of the subsequent experiment. They were introduced to the dynamic rating method using the trackball and the sliding bar, and were instructed to also rate the average risk and speed after each video. Additionally, participants were asked to minimize the movement of their head while watching a video to reduce motion artifacts. After going through all explanations and instructions, participants entered the scanning room, and additional care was taken to provide a trackball to the participants' right hands before scanning. A T1 anatomical scan was recorded, after which the experimenter checked on the participant and informed them that the experiment was about to begin. The experiment began with 30 s of baseline, followed by four 2‐min videos presented in counter‐balanced order, which participants first rated for dynamic risk and then once more for average risk within 20 s after the end of each video. After the first block, the experimenter checked once more on the participant and asked them to start the second block, which followed the same procedure while participants rated the videos for speed. In total, one block consisted of a 30‐s baseline period, 8 min of video presentation, and 80 s of post‐task questionnaires; one block took thus 9 min and 50 s to complete, and the whole experiment took 19 min and 40 s.

### 2.5 Data acquisition

A SIEMENS Magnetom Prisma 3 T scanner was used to acquire MRI data (Siemens Medical Systems, Erlangen, Germany) with a 20‐channel SENSE head coil and a 64‐channel neck coil (Center for Neuroscience Imaging Research, Sungkyunkwan University, Suwon, South Korea). Structural MRI images were collected using a T1‐weighted sagittal high‐resolution MPRAGE sequence (repetition time (TR) = 2300 ms, echo time (TE) = 2.28 ms, flip angle (FA) = 8°, field of view (FoV) = 256 mm, matrix size: 256 × 256, voxel size = 1 × 1 × 1 mm^3^, 192 axial slices). Functional imaging was performed using a gapless echo‐planar imaging (EPI) sequence and integrated parallel acquisition techniques (iPAT) applied for slice acceleration (TR = 2000 ms, TE = 30 ms, flip angle (FA) = 90°, field of view (FoV) = 200 mm, matrix size: 100 × 100, voxel size = 2 × 2 × 2 mm^3^, 72 axial slices). Since each session lasted for exactly 9 min and 50 s (excluding dummy volumes), the total number of recorded volumes for each session was 295.

### 2.6 Imaging data preprocessing

Data were preprocessed using SPM12 (Wellcome Trust Centre for Neuroimaging, London, UK; http://www.fil.ion.ucl.ac.uk/spm/). First, all volumes were realigned to the initial volume and checked for excessive head translation or rotation; none of our participants exceeded the threshold of 2 mm/TR. After realignment, the T1 image was co‐registered with the mean EPI and tissue segmentation was performed using the segment function in SPM. Functional data were then normalized into MNI space and to 2 × 2 × 2 mm^3^ using the normalize function, followed by smoothing with a Gaussian kernel of 8‐mm FWHM.

### 2.7 Behavioral data analysis

For the behavioral data and correlational searchlight analyses, we averaged the dynamic ratings over all frames to acquire the average dynamic ratings for each video. Second, we applied repeated‐measures ANOVAs to the average dynamic ratings as well as the post‐video ratings to assess subjective differences in experience across videos, and additionally conducted post hoc analyses to investigate perception differences between videos. Finally, we averaged the dynamic ratings across every 2 s for later use in the sliding window correlation analysis.

### 2.8 fMRI data analysis

The main goal of the present study was to identify whole‐brain activation correlated with dynamic perceptions. To attain this, we first used a general linear model (GLM) to detect participants' video‐specific neural activity as well as neural activity during each volume. Since participants watched four different videos in two different blocks, the video‐specific GLM contained four variables per session with six head‐motion‐related covariates, yielding four beta estimates for each voxel in each session (similar to (Ju & Wallraven, 2019)). These beta estimates were not analyzed per se but formed the basis for the correlational searchlight analyses, as explained below. Furthermore, each video was 2 min long and each volume lasted 2 s, resulting in 60 volumes per video. We applied the GLM to each TR while participants performed tasks of real‐time ratings on risk and speed and also applied SPM12 default high‐pass filtering to address scanner drift. This process resulted in 60 beta estimates, representing the task‐related influence on the brain's BOLD signal, for each video. Consequently, with the four videos and risk and speed rating conditions, we obtained a total of 480 beta estimates per participant. These were used in the sliding window analysis to investigate correlations between dynamic perception and whole‐brain activation.

### 2.9 Correlational searchlight analysis

Next, we conducted a multi‐voxel‐based correlational analysis to investigate associations between overall brain activation and subjective risk and speed perceptions. To detect brain regions associated with overall risk and speed perceptions, we performed multivariate, whole‐brain correlational searchlight analyses (Bulthé et al., 2014; Op de Beeck et al., 2008) using the beta estimate from each video using GLM and the subjective risk and speed ratings from the post‐task questionnaire and average dynamic ratings. We first averaged the post‐task risk and speed ratings across all participants for each video, and then computed the differences in these average ratings between each pair of videos. This resulted in a 4 × 4 behavioral difference matrix A, where each entry represented the difference in average risk and speed ratings between the two videos. We then averaged the average dynamic ratings, across participants and for each video, to create a 4 × 4 behavioral difference matrix *B* where each cell contained average behavioral ratings across different videos. A similar procedure was applied to the beta estimates averaged within an 8‐mm radius (corresponding to a 64‐voxel volume), yielding the 4 × 4 neural activity difference matrix *C*. Correlations between *A* and *B* as well as *A* and *C* were obtained for every voxel and every participant, and the resulting p‐values were subjected to a false discovery rate (FDR) adjustment to determine significant voxels at the group level.

### 2.10 Sliding window based correlational analysis

**FIGURE 2:**
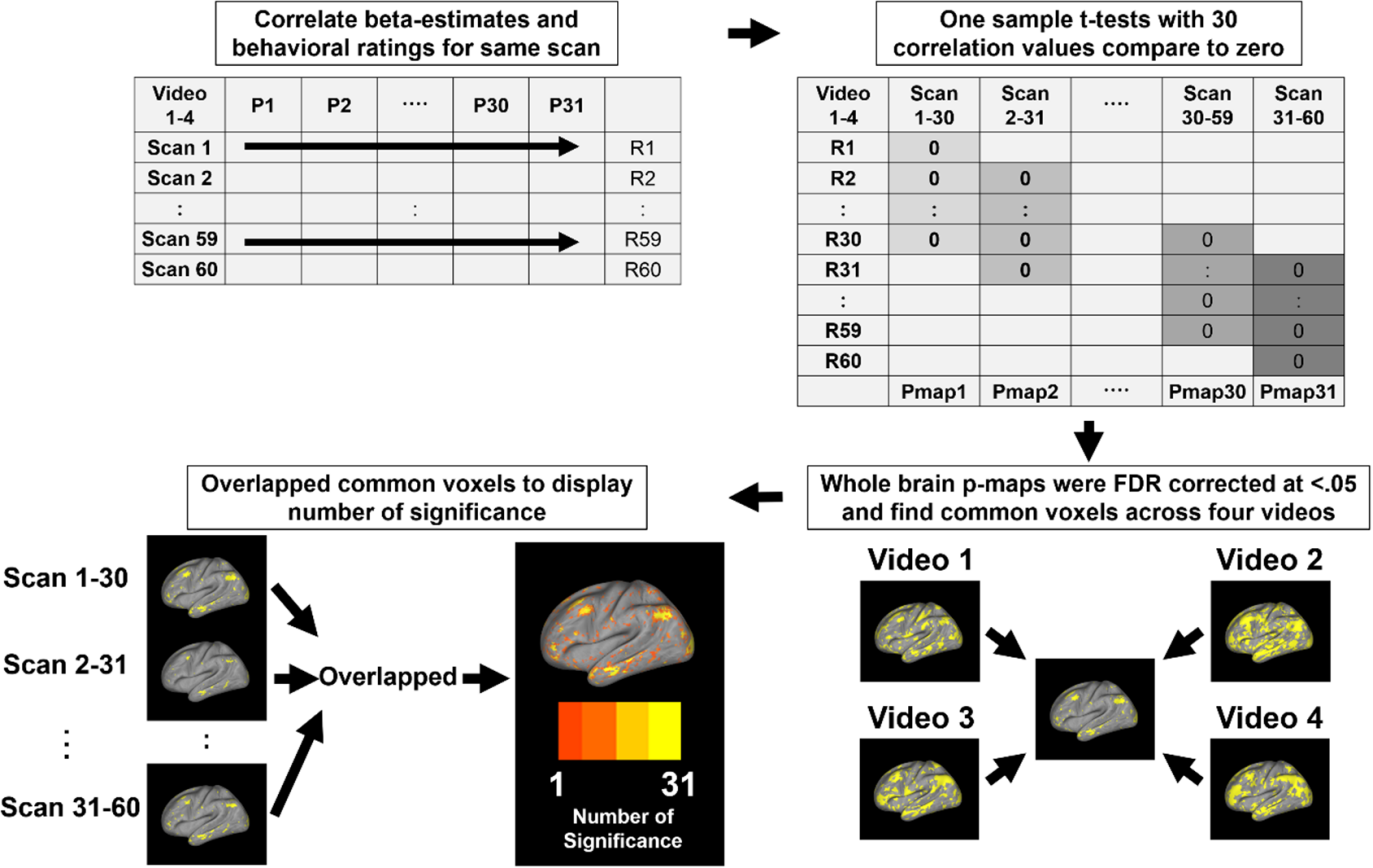
Correlation analysis pipeline to decode dynamic risk and speed perceptions.

Next, we performed the main analysis of this study, to identify associations between dynamic perceptions and participants' whole‐brain activation (as defined above). Since all participants watched the same video, we first analyzed pair‐wise correlations between the dynamic behavioral ratings and beta estimates, across participants and for all volumes. We obtained 60 volumes as well as 60 whole‐brain correlation maps for each video. Next, based on the whole‐brain correlation maps, we created a sliding window of size 30 (including 30 volumes and with a length of 1 min, based on previous studies showing that empirically, window sizes of 30–60 s produce robust results for investigations of cognitive states Hutchison et al., 2013; Shirer et al., 2011) to investigate whole‐brain activation changes by shifting from window to window. We used 30 whole‐brain correlation maps as one group and performed a second‐level analysis to determine whether the 30 correlation values were significantly positive or negative compared to the zero value based on one‐sample t‐tests. We then entered the whole‐brain p‐map into the standard FDR procedure to identify significant voxels for each video. Next, we extracted common significant voxels across all four videos to identify brain regions that were significantly correlated with dynamic risk and speed for one sliding window. We repeated the procedure after shifting the sliding window, thereby obtaining 31 whole‐brain activation maps in total (volumes from 1–30 to 31–60) for risk and speed. Finally, we overlapped all whole‐brain activation maps to display the significance of each voxel (see Figure 2 for the overall analysis process). In addition, we investigated whole‐brain activation changes associated with subjective risk and speed perceptions across time using whole‐brain activation map changes induced by window shifting. We divided the sliding window into four quarters (Hutchison et al., 2013; Shirer et al., 2011) and set the minimum length to 30 s, based on previously established methodology (Mokhtari et al., 2019): the first quarter started at time points 1–8, the second at time points 9–16, the third at time points 17–24, and the fourth at time points 25–31.

### 2.11 Decoding functional correlates of risk and speed perception

Finally, to interpret the whole‐brain activation maps at the system level, we investigated the functional correlates of risk and speed perception based on an overlapped activation map derived from the dynamic correlation analysis (as shown in Figure 2) that we entered into the Neurosynth decoding function (Yarkoni et al., 2011) (http://neurosynth.org/decode) to find functional terms related to the surviving voxels that used all survived voxels to find most the likely correlated functional terms. The Neurosynth decoder tool enables meta‐analyses to automatically extract functional terms corresponding to mask—at the time of writing (2023‐02‐17), the database lists 1334 terms reported in 14,371 studies. To identify the most likely functional associations of subjective risk and speed perception, we extracted the ten highest‐correlated nonanatomical terms extracted by Neurosynth. We then used these ten features as target terms to assess changes in functional correlates derived from the 31 activation maps created through the window shifting procedure and entered them into the Neurosynth decoding function.

## 3 RESULTS

### 3.1 Behavioral data

**FIGURE 3:**
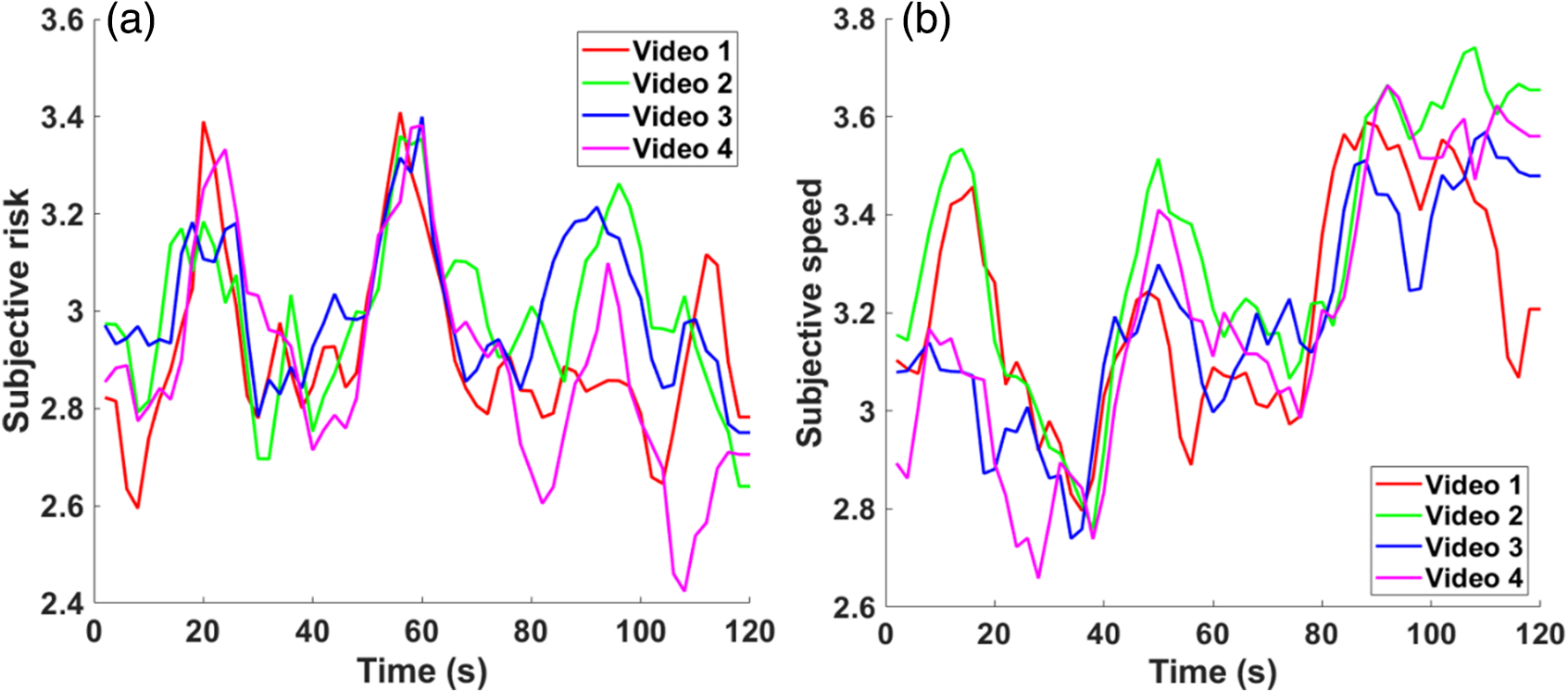
Average dynamic rating across participants over time (a) risk (b) speed.

Based on the results of our evaluation experiment, we initially selected the four videos with the most extreme speed/risk values (see Supporting information, Tables S2 and S3 for detailed methods and results). Subsequently, we analyzed the behavioral data collected during the fMRI experiment, based on the dynamic and post‐task ratings. First, we calculated the number of rating changes in dynamic ratings to confirm participants dynamically change their ratings. The results revealed that in dynamic risk rating, average number of rating change was 34.91 (SD = 10.96) and in speed rating, average number of rating change was 30.90 (SD = 10.66). These findings confirmed that participants dynamically changed their ratings during the task. Second, we investigated the changes in dynamic behavioral ratings across the four videos, both in terms of speed and risk rating conditions. The results demonstrated a synchrony in ratings for both speed and risk, indicating that the same driving course evokes similar subjective perceptions (see Figure 3). Third, we performed a correlation analysis between the average dynamic and post‐task ratings to confirm the reliability of the ratings across rating methods (see supporting information, Figure S2 for correlation analysis between different rating methods). We found that ratings were significantly correlated with both risk (*r* = 0.60, *p* < .001) and speed perception (*r* = 0.40, *p* < .001), confirming that average dynamic ratings and post‐task ratings were similar across participants. Next, we applied a repeated‐measures ANOVA to the average dynamic and post‐task ratings to compare subjective perception differences between videos (see Table 1). The analysis yielded significant differences between overall risk and speed perception in post‐task ratings (risk rating: F(3,28) = 6.426, *p* = .001; speed rating: F(3,28) = 10.935, *p* < .001), indicating overall risk and speed perception differences across different videos but no significant differences in average dynamic ratings (average dynamic rating‐based risk rating: F(3,28) = 2.606, *p* = .057; speed: F(3,28) = 1.294, *p* = .281). Next, we performed a post hoc analysis on the post‐task ratings to investigate individual perception differences between videos and found that speed ratings for Videos 1 and 2 were significantly higher than those for Videos 3 and 4 (Video 1–Video 3: t(30) = 3.14, p = .004; Video1–Video 4: t(30) = 4.42, *p* < .001; Video 2–Video 3: t(30) = 3.93, *p* < .001; Video 2–Video 4: t(30) = 4.17, *p* < .001), whereas risk perception was only significantly lower for Video 4 compared to Videos 1 and 2 (Video 4–Video 1: t(30) = 4.15, *p* < .001; Video 4–Video 2: t(30) = 2.76, *p* = .01) (see details in Table S4). Finally, we investigated the potential influence of sex on speed and risk perception, and found no significant differences between the sexes (see details in supporting results and Table S5). In summary, our study revealed significant differences in perception across videos in post‐task ratings. However, no significant differences were found in dynamic ratings, which indicates that post‐task ratings and average dynamic ratings represent different aspects of subjective perception in the videos.

**TABLE 1:** Behavioral ratings for the four videos used in the main experiment.

	Video 1	Video 2	Video 3	Video 4
Average risk (SD)	3.25 (0.72)	3.03 (0.64)	3.11 (0.81)	2.73 (0.79)
Average speed (SD)	3.49 (0.62)	3.68 (0.68)	3.13 (0.68)	2.96 (0.81)
Dynamic average risk (SD)	2.98 (0.41)	3.00 (0.42)	3.09 (0.43)	2.89 (0.39)
Dynamic average speed (SD)	3.13 (0.43)	3.26 (0.34)	3.12 (0.42)	3.11 (0.44)

Abbreviations: SD, standard deviation.

### 3.2 Results from correlational searchlight analysis

**FIGURE 4:**
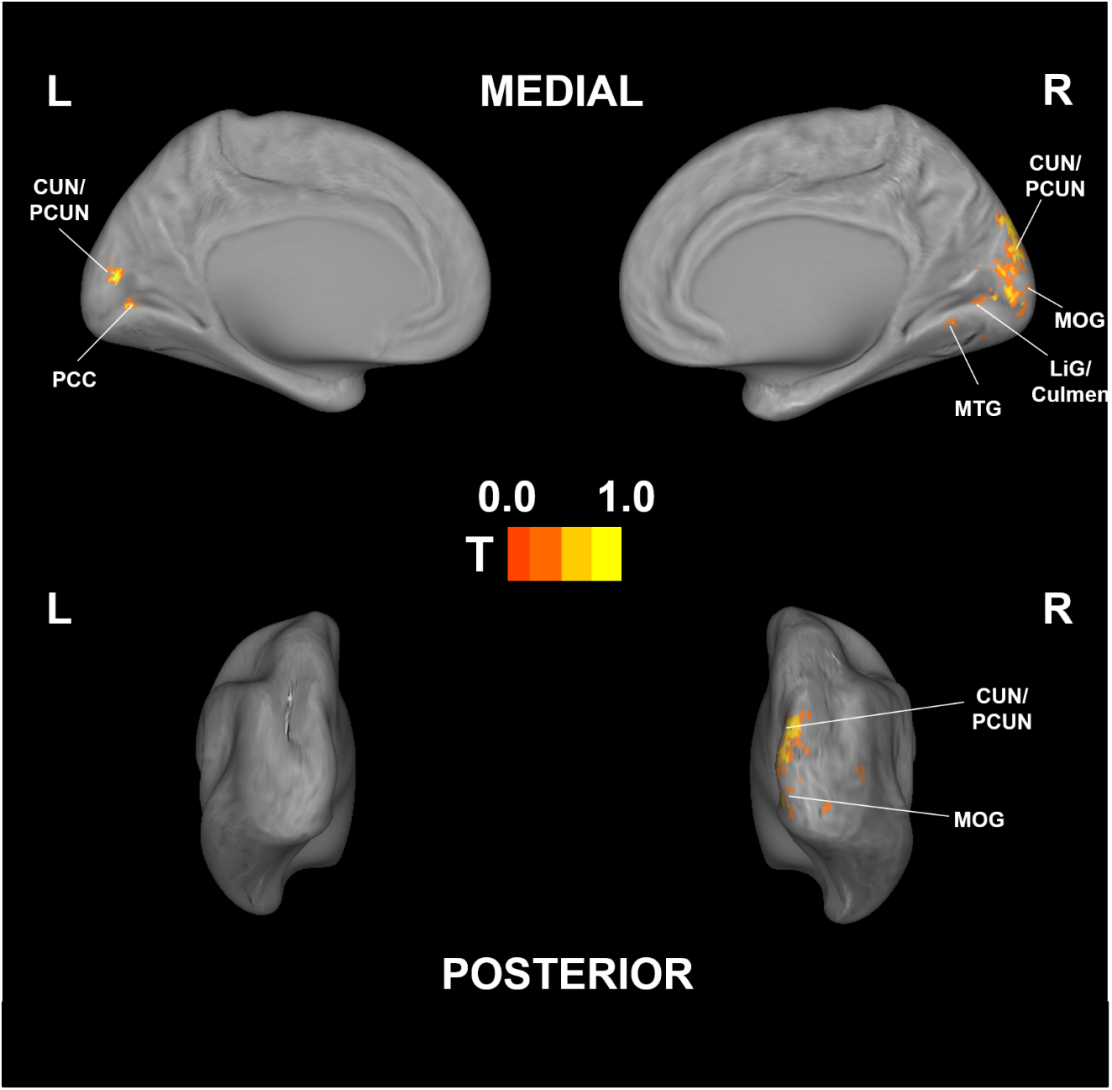
Correlational searchlight analysis for brain regions decoding post‐video risk ratings. Results are *p* < .001 uncorrected for visualization. All t‐values are rescaled to 0–1 for better comparison. CUN: cuneus, PCUN: precuneus, PCC: Posterior cingulate, MOG: middle occipital gyrus, LiG: lingual gyrus, MTG: middle temporal gyrus.

Before analyzing the dynamic perception data, we first performed a correlational searchlight analysis to identify brain regions correlated with overall risk and speed based on the post‐task ratings (See supporting information Figure S3 and S4 for univariate analysis results of risk and speed rating for all videos). We found significant correlations for risk perception in visual‐associated regions, including the cuneus, lingual gyrus, and middle occipital gyrus (see Figure 4), as well as in the middle temporal gyrus, posterior cingulate, and subgyrus (see Table 2). The same analysis applied to the average dynamic ratings detected similar regional activation for risk perception (see Supporting information, Figure S5, and Table S6 for details). However, we found no voxels showing significant activation for speed perception, neither in the post‐video nor in the average dynamic ratings, which indicates that the conventional searchlight analysis was not able to find associated brain regions.

**TABLE 2:** Location information of the correlational searchlight analysis.

Region (AAL)	Peak voxel	*Z*‐score	Number of voxels
Culmen	8, −64, −6	3.91	10
Cuneus	8, −88, 32	5.97	444
Lingual gyrus	8, −64, −4	4.22	22
Middle occipital gyrus	24, −90, 2	4.69	50
Middle temporal gyrus	58, 4, −20	3.81	18
Posterior cingulate	−2, −50, 14	3.96	13
Precuneus	−4, −82, 8	4.93	18
Subgyrus	−18, −12, 58	4.15	21

### 3.3 Results from sliding window based correlational analysis

**FIGURE 5:**
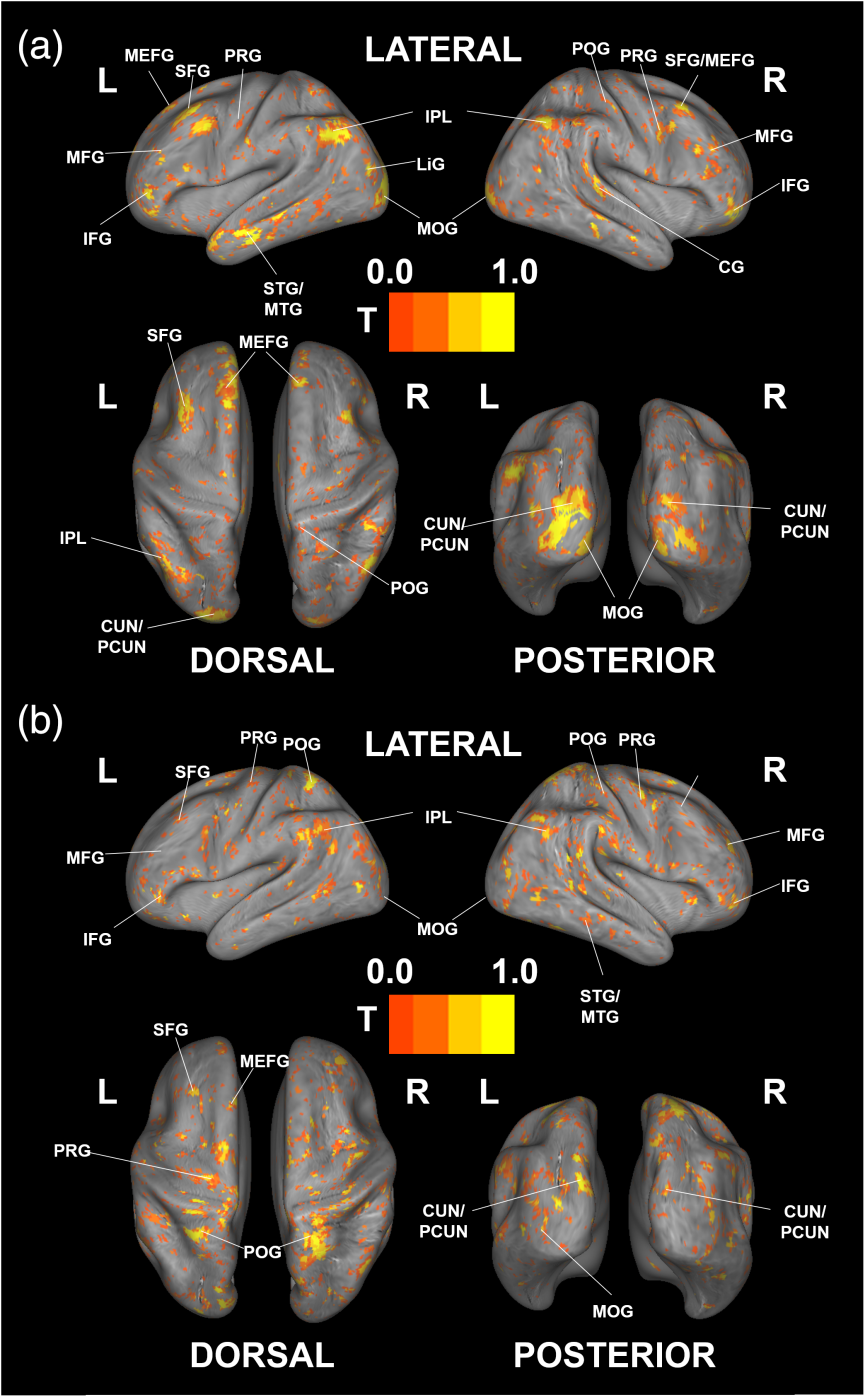
Sliding window‐based correlation analysis of risk and speed perception (*p* < .05 FDR‐corrected). (a) Risk perception. (b) Speed perception. Values are rescaled to 0–1 to display frequencies of significance from 1–30 to 31–60 volumes. MEFG: Medial frontal gyrus, SFG: superior frontal gyrus, MFG: middle frontal gyrus, IFG: inferior frontal gyrus, IPL: inferior parietal lobule, LiG: lingual gyrus, CUN: cuneus, PCUN: Precuneus, PoG: postcentral gyrus, PRG: precentral gyrus, STG: superior temporal gyrus, MTG: middle temporal gyrus, MOG: middle occipital gyrus.

To determine which brain regions correlate with dynamic behavioral ratings, we performed a sliding window‐based correlation analysis on the dynamic risk and speed rating data (results were FDR‐corrected at *p* < .05): significant regions were first identified for each video and each sliding window, and significant voxels across the four videos for the same sliding window were extracted to determine common brain activation associated with risk and speed. We then overlapped all brain activation maps and displayed the frequency of significance for each voxel. Figure 5‐A visualizes the results for dynamic risk, showing widespread brain activation mainly in the frontal, occipital, and temporal regions of the brain, including the inferior, medial, middle, and superior frontal gyrus, as well as the cuneus, precuneus, lingual gyrus, middle occipital gyrus, and middle and superior temporal gyri. The dorsal part of the brain was identified as an additional associated region, including the inferior parietal lobule, pre‐ and postcentral gyri, and cingulate gyrus (see Table 3). Next, our analyses showed that dynamic speed mainly activated the dorsal and frontal parts of the brain, including the precentral and postcentral gyrus, inferior parietal lobule, and middle, medial, inferior, and superior frontal gyrus. Additional activation was observed in the temporal and occipital regions of the brain, including the middle occipital gyrus, precuneus, cuneus, and middle and superior temporal gyrus (see Figure 5B and Table 3). A comparison yielded greater activation for risk perception in the frontal and occipital parts of the brain, while speed perception correlated with more activation in motor‐related regions; the common brain activation was weak and widely distributed, indicating that different processes are involved in the decoding of risk and speed (see Supporting information, Figure S6, and Table S7 for details). These results remained consistent when all volumes were used as a sliding window for a correlation analysis on dynamic risk and speed (see Supporting information, Figures S7–S9, and Table S8 for details).

**TABLE 3:** Location information of the dynamic rating‐based correlation analysis.

Risk	Region (AAL)	Number of voxels
	Cingulate gyrus	573
	Cuneus	1335
	Inferior frontal gyrus	1107
	Inferior parietal lobule	1215
	Lingual gyrus	687
	Medial frontal gyrus	866
	Middle frontal gyrus	2184
	Middle occipital gyrus	981
	Middle temporal gyrus	1477
	Postcentral gyrus	760
	Precentral gyrus	707
	Precuneus	850
	Superior frontal gyrus	1807
	Superior temporal gyrus	900
Speed	Cuneus	650
	Inferior frontal gyrus	807
	Inferior parietal lobule	978
	Medial frontal gyrus	949
	Middle frontal gyrus	1776
	Middle occipital gyrus	508
	Middle temporal gyrus	1170
	Postcentral gyrus	1578
	Precentral gyrus	1257
	Precuneus	1039
	Superior frontal gyrus	1255
	Superior temporal gyrus	898

*Note*: All analyses are *p* < .05, FDR‐corrected, and reported clusters have more than 500 significant voxels.

Abbreviation: AAL, Automated Anatomical Labeling.

**FIGURE 6:**
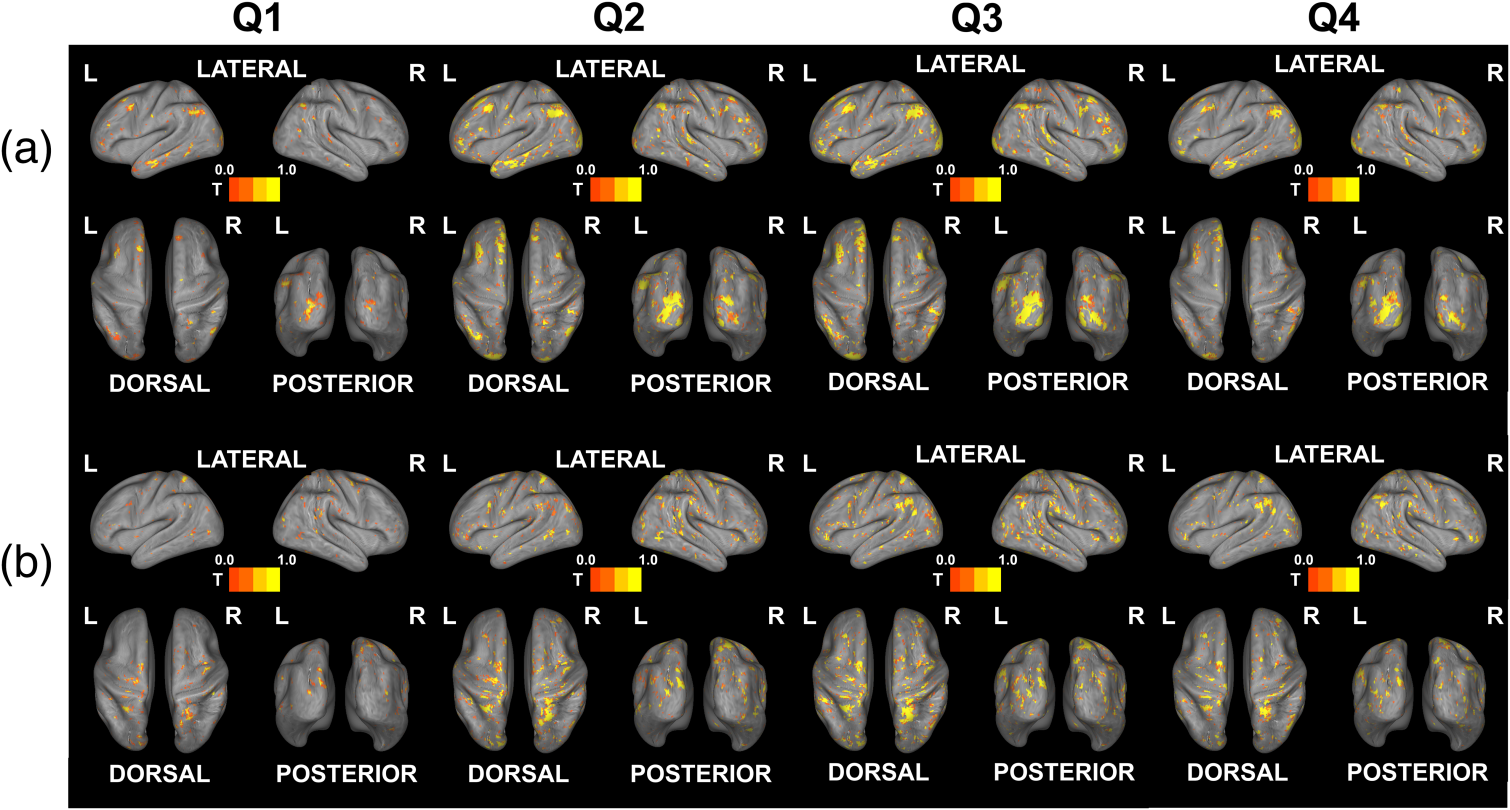
Sliding window‐based correlation between beta estimates and dynamic behavioral ratings divided into four quarters (*p* < .05 FDR‐corrected). (a) Risk perception. (b) Speed perception. Q1: **volume** 1–30 to 8–37, Q2: **volume** 9–38 to 16–48, Q3: **volume** 17–46 to 24–53, Q4: **volume** 25–54 to 31–60.

Next, we divided the sliding window into four quarters and assessed changes in whole‐brain activation induced by window shifting. We found that for both risk and speed perception, the second and third quarters of sliding windows showed the highest activations (see Figure 6; the activation numbers for the sliding windows are displayed in Figure S10). For risk perception, the last quarter of the sliding window showed a large decrease in frontal region activation compared to occipital region activation (see Figure 6A, Q4). In contrast, for speed perception, the dorsal parts of the brain showed the highest contribution to the observed activation in all quarters (see Figure 6B).

### 3.4 Decoding functional correlates of dynamic perception

**FIGURE 7:**
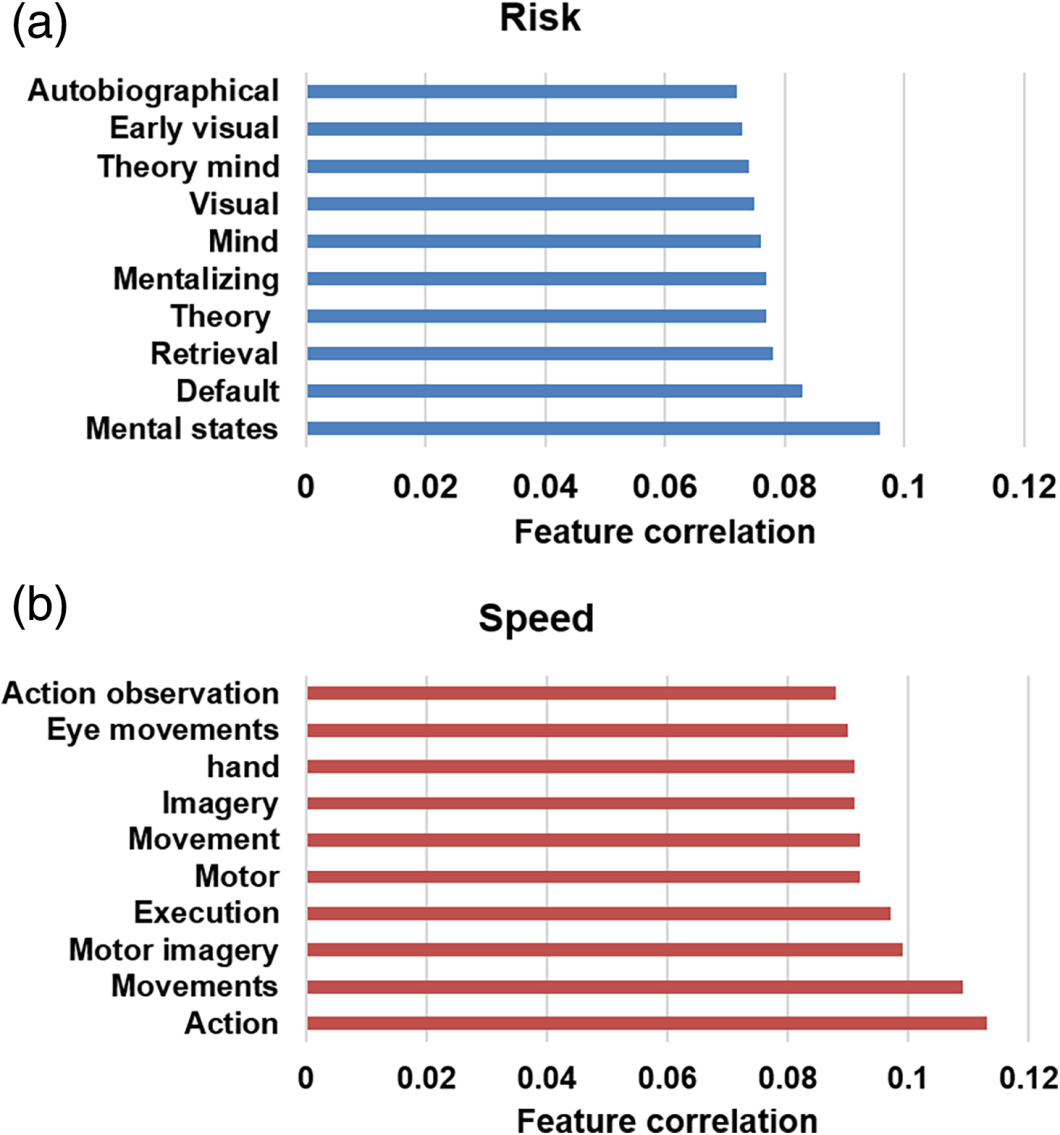
Psychological features associated with the overall dynamic ratings displayed in Figure 5, extracted using the decoding function of Neurosynth. (a) Risk perception (b) Speed perception.

**FIGURE 8:**
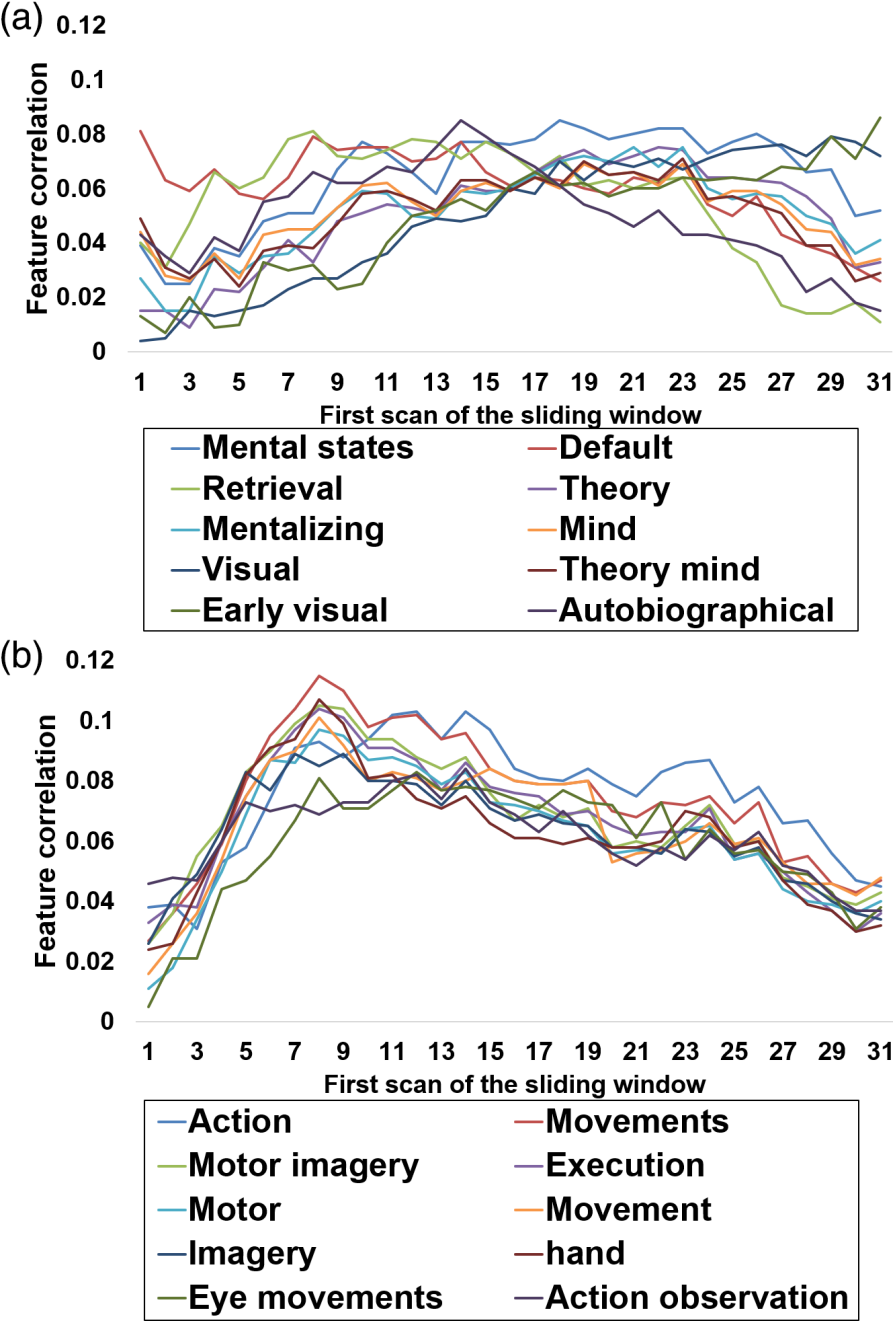
Psychological features associated with the dynamic ratings from sliding window 1–30 to 31–60, extracted using the decoding function of Neurosynth. (a) Risk perception. (b) Speed perception. The term ‘Theory’ is associated with ‘Theory of mind’ and ‘default’ is associated with ‘Default mode network’.

We used Neurosynth to decode feature correlations for dynamic risk and speed perceptions to help interpret the complex whole‐brain activation maps acquired in the prior analysis. For this purpose, we extracted the activation map from Figure 5 and decoded the surviving voxels using the Neurosynth meta‐analysis map. We found that significant correlations of risk were mainly associated with mental states, mind, visual aspects, as well as task, memory, and self‐related aspects such as default, retrieval, and autobiographical features (see Figure 7A), while speed perception mainly correlated with movements, motor, eye, action, and execution aspects (see Figure 7B). We then shifted the sliding window to investigate functional correlate changes from volume 1–30 to 31–60 for both risk and speed perception, using the ten functional terms derived from Figure 7. We found that for dynamic risk perception, *default* showed the highest feature correlation in the first part of the volume, while *retrieval*, *autobiographical*, *mental states, visual*, and *early visual* showed the highest feature correlations when the sliding window changed from 1–30 to 31–60. In contrast, for dynamic speed perception, *action, movement*, and similar terms such as *action observation* showed the highest feature correlations for the overall sliding window (see Figure 8). In short, feature correlations for risk dynamically changed over time, while those for speed remained consistent (the same analysis on anatomical terms is described in Supporting information, Figures S11 and S12).

## 4 DISCUSSION

In the present study, first‐perspective driving videos and dynamic rating methods were used to investigate the neural correlates of dynamic risk and speed perceptions. Importantly, a dynamic rating‐based correlation analysis showed that broad areas of the brain contributed to the decoding of subjective risk and perception, which was not found in a correlational searchlight analysis. In addition, a dynamic rating‐based analysis showed that risk perception was mainly decoded in the frontal, occipital, and temporal parts of the brain, while speed perception was mainly decoded in the dorsal parts of the brain. An additional feature correlation analysis showed that mental and mind process‐related brain regions decode risk while motor and movement‐related brain regions decode speed.

### 4.1 Behavioral differences across videos

We used different driving videos taken on the same racing course to induce different risk and speed perceptions. Our post‐task questionnaire results indicated substantial differences in risk and speed perceptions, which may be associated with other cars driving on the same course, per the following: First, perceived risk usually increases when the distance to a preceding car decreases and decreases when the distance to a preceding car increases (Kondoh et al., 2008). This was demonstrated in participant responses to Videos 1 and 3 in our experiment, in which the driver maintained a short distance from the preceding car for a longer time than in Videos 2 and 4. Second, perceived speed increased when the car was driving straight or overtook other cars and decreased when the car was driving in curves or when the distance to the car in front increased. Our Videos 1 and 2, which were recorded at a higher speed and contain a higher number of overtakes than Videos 3 and 4, demonstrate this.

Importantly, these differences were observed only in the post‐task questionnaire but not in the average dynamic ratings. A potential reason for this discrepancy is that online and offline evaluations of behavioral ratings generally vary. For example, previous studies show that research paper recommendation systems based on online and offline evaluations sometimes contradict each other, and several studies show significant differences between online and offline evaluation results (Beel et al., 2015; Beel & Langer, 2015). Moreover, previous studies comparing online verbal reports and offline questionnaire results show differences in neural activity, implying that real‐time and post‐task measurements are processed differently at the neural level (Durning et al., 2013). Our dynamic evaluation did not consider overall perception and assessed only risk and speed perception changes during the currently watched videos. Potential perception differences across videos were not assessed and differences in risk and speed perception across videos could, therefore, not be revealed.

### 4.2 Decoding perception through searchlight analysis

In the second part of our analysis, we conducted a correlational searchlight analysis to decode risk and speed perceptions based on post‐video ratings. We found that only risk perceptions significantly correlated with brain activation, detected in the occipital regions, as well as in a small portion of the middle temporal and cingulate regions of the brain. These findings support previous studies showing that vision‐related regions (Gharib et al., 2020; Hirth et al., 2007), the middle temporal region (Megías et al., 2018), and the posterior cingulate cortex (Qin et al., 2009) are associated with subjective risk perceptions and imply that visual input, such as the distance from a preceding car, influences overall risk perception. Interestingly, when we performed the same analysis on the average dynamic risk data, a similar association with brain activity was observed (see Figure S5, Table S6), demonstrating that average risk ratings also reflected overall perception, since there were no critical situations in our videos that could have been perceived as dangerous. In contrast, the correlational searchlight analysis yielded no significant associations with speed perception for either rating method. A potential reason for this is that average speed ratings cannot reflect rapid changes in speed while driving. This is confirmed by the fact that the difference in minimum and maximum driving speeds was above 100 km/h in all videos and that the overall speed perception ratings were higher than the risk ratings (see Table S4). In short, our correlational searchlight analysis partially supported the first hypothesis of this study that revealed only a limited area activation for decoding subjective perceptions, and significant activation was detected only for average dynamic ratings of risk, which implies that conventional correlational searchlight analyses have a limited ability to decode perceptions that rapidly change over time.

### 4.3 Sliding window‐based correlation analysis

We also conducted a sliding window‐based analysis to investigate which regions contribute to the decoding of dynamic risk and speed. We found that mainly frontal, occipital, and temporal parts of the brain decode risk while dorsal parts decode speed.

First, concerning dynamic risk, we found correlations with vision‐related regions as well as with frontal and temporal parts of the brain. These findings are consistent with a previous meta‐analysis which showed that vision‐related regions are associated with decision risk (Mohr et al., 2010). Further supporting this, studies on driving behavior have shown that activation in the occipital gyrus increases linearly with an increase in risk levels (Megías et al., 2018), and that hazard perception is associated with occipital region activation (Gharib et al., 2020; Hirth et al., 2007). Additionally, vision‐related activation was also detected in our searchlight analysis, supporting the second hypothesis of the study, indicating that the visual system contributes to decoding dynamic risk. The involvement of the frontal regions in risk decoding can be explained by the function of the medial and inferior frontal gyrus. Previous studies have found that the dorsal medial prefrontal cortex (DMPFC) is activated during anticipated risk in tasks involving economic decisions (Mohr et al., 2010; van Duijvenvoorde et al., 2015), and the inferior frontal gyrus is a key region for perceived risk in both economic decision‐making (Fukunaga et al., 2018) and in driving (Megías et al., 2018). This aligns with the activation patterns detected in the present study. Finally, the temporal region association with risk perceptions is also supported by previous research showing that the activity of the middle and superior temporal gyrus increases with risk level in driving task (Megías et al., 2018) and that the superior temporal gyrus is associated with risk anticipation in general (Mohr et al., 2010). Interestingly, when we divided the sliding window into four quarters and analyzed changes in activation compared to the vision‐related areas, we found that the contribution of the frontal regions decreased in the final quarter. These findings suggest that the key regions decoding risk shift from the frontal to the occipital parts of the brain over time, and that evaluation strategies are shifting from risk prediction to risk‐level evaluation based on experiences.

Second, regarding dynamic speed, we found a correlation with mainly the dorsal visual stream of the brain, including motor, visual, and frontal areas. This is in line with previous research that found that the dorsal visual stream is associated with an egocentric view (Goodale & Milner, 1992) and that the role of the dorsal visual stream is motor programming (Milner & Goodale, 2008). Since our experimental design requires participants to rate the videos from an egocentric perspective, it is plausible to assume that the dorsal visual stream plays a role in decoding speed.

We found additional activation in the premotor areas and superior temporal and middle temporal regions for speed perception, which is supports previous research showing that premotor areas and superior temporal regions are needed for biological motion perception (Saygin, 2007; van Kemenade et al., 2012) and that the middle temporal regions are associated with visual motion perception in general (Kaderali et al., 2015; Takemura et al., 2012). However, in contrast to risk perception, the areas involved in decoding speed perception did not change throughout the task, implying that participants used only one consistent strategy to evaluate speed.

Interestingly, the largest brain activation was observed in the middle of the shifting sliding window for both risk and speed. This suggests that the brain may require time to select the optimal strategy for evaluating perceptions and that long assessments may reduce decoding efficacy. Additionally, this finding suggests another explanation for restricted brain activation in conventional searchlight analyses, as average neural activity cannot reflect changes in evaluation strategies or a reduction in decoding effectiveness. Overall, in contrast to what the conventional searchlight analysis suggested, our sliding window‐based correlation analysis reveals that broad areas of the brain contribute to decoding perception and that their contribution fluctuates over time, supporting the third hypothesis of the study.

### 4.4 Decoding functional correlates of dynamic perception

We next used a meta‐analysis brain map database to investigate the functional correlates of changes in risk and speed across shifting sliding windows. For risk perception, *default* and *memory retrieval* showed the highest feature correlations in the first quarter, *mental states* and *theory of mind* in the second and third quarters, and vision‐related terms in the fourth quarter. These findings imply that participants began to construct an assessment strategy and retrieved information from memory to assess the scene during the first three quarters of the sliding window. Participants then assessed the driving risk based on their assumptions about the intentions of other drivers, and in the last quarter, they defined their subjective risk levels using visual input. In summary, the main brain regions decoding risk perception changed over time, which is also supported by our previous analysis of anatomical regions (also see Figure S10 for the anatomical terms derived from the decoding analysis).

Second, our analysis of speed perception showed that the terms *movement* and *action* showed the highest feature correlations across all windows. These overall consistent feature correlations imply that after participants set up a strategy to evaluate speed, they maintained their strategy over the course of the video. This is also supported by our findings that the premotor and parietal regions showed the highest contribution across time (also see Figure S10 for the decoding results of anatomical terms). In short, our decoding of functional correlates again confirms that the brain regions that decode risk perceptions change over time, supporting the last hypothesis of the study, while those that decode speed perceptions are consistent.

### 4.5 Limitations

This study has certain limitations that should be addressed in future research. First, all participants performed the risk perception task before the speed perception task, which might have led to order effects. However, the speed perception task can provide participants with feedback on risk, since risk perception is influenced by order, and assessing risk second order decreases risk assessments (Buratti & Allwood, 2019), and rate speed before risk may affect subjective risk. We therefore presented the tasks in a fixed order, assuming that the effect of order on speed is relatively small. Future work should take such order effects and their potential influence into account. Second, the results of our dynamic ratings in the main experiment differed from the evaluation study ratings. A potential explanation for this discrepancy is that participants were asked to minimize their head movements during the fMRI experiment, which may have resulted in small changes in ratings. This is supported by a previous fMRI study showing that greater head motion is associated with poor performance on inhibition tasks in older adults (Hausman et al., 2022). In our study, rating changes were similar between the fMRI and the evaluation experiment, but maximum ratings were significantly lower in the former (see Table S4). This implies that participants might have restricted their movements during scanning. Although the dynamic evaluations showed no such differences, the post‐video ratings yielded similar results. In order to test the influence of head movements on subjective ratings, in future research, behavioral studies should restrict participants so that results can be compared to fMRI findings; for example, eye‐tracking devices with a chin rest can minimize head movements outside the scanner and further enhance the accuracy of dynamic perception measurements. Third, potential delays in hemodynamic response might compromise the accuracy of dynamic correlational analysis. However, in our study, no specific events such as accidents or emergency braking occurred, and hemodynamic responses did not significantly change during the task. This suggests that applying time derivative models for corrections would be inappropriate. Additionally, we employed a sliding window‐based correlational analysis for dynamic ratings, effectively compensating for any hemodynamic response delays, provided participants did not alter their ratings with each volume. On average, participants took about 4 s to change their ratings, indicating that they consistently maintained the same rating during these intervals, potentially mitigating the issues related to hemodynamic delay. Additionally, research by Polimeni et al (Polimeni & Lewis, 2021) suggested that the onset of the BOLD response occurs almost instantaneously following neuronal activity, indicating that valuable neuronal information can be extracted from the initial stages of the BOLD response to identify brain regions associated with dynamic perception changes. Finally, in our study, participants only evaluated risk and speed, without engaging in actual risk‐taking or increasing speed. This led to different brain region activations compared to previous risk‐taking studies. For example, several financial risk‐taking studies have shown association of anterior insula with risk‐taking behaviors (Häusler et al., 2018; Kuhnen & Knutson, 2005), a connection not observed in our study. The lack of significant association with the anterior insula in our study could be attributed to the distinction between risk perception and actual risk‐taking, as participants were not required to engage in real risks. This distinction also accounts for the absence of sex differences in risk and speed perceptions in our study, corroborated by prior road safety attitude research indicating that although male participants were less concerned about accidents compared to females, there were no significant differences in perceived risk (Cordellieri et al., 2016). We expect that future research should implement driving simulations that require participants to take risks while driving. Additionally, using verbal reports or post‐task video recordings could help confirm differences in brain regions associated with risk perception, speed perception and risk‐taking, and speeding behavior during driving.

## 5 CONCLUSION

To the best of our knowledge, the present study is the first to decode the dynamic aspects of subjective risk and speed with the goal of identifying related brain networks. Our dynamic correlation analysis detected frontal, visual, and temporal networks of brain areas associated with risk perception, and determined that the dorsal visual stream is associated with perceived speed. The decoding of functional correlates of subjective risk and speed perceptions described here highlights the potential of our experimental paradigm to decode dynamic subjective experiences. Previous studies have found that risk perception directly influences attitudes towards road safety (Ram & Chand, 2016). It was found that individuals involved in more than three accidents perceived driving on roads as less risky (Ngueutsa & Kouabenan, 2017). Furthermore, a previous study showed that poor speed perception, both of one's own vehicle and of vehicles approaching from behind, increases the risk of motor vehicle accidents (Čubranić‐Dobrodolac et al., 2022). This suggests that inaccurate perceptions of both risk and speed critically influences road safety. We believe that the present study is an initial step towards understanding the dynamics of risk and speed perception in driving, that can be used to predict potential accidents caused by incorrect estimations of risk and speed.

Additionally, we expect that future studies will use similar paradigms to decode dynamic perception or cognition to shed more light on how the brain dynamically decodes information. For example, it would be interesting to correlate dynamic functional connectivity with dynamic experiences to provide another view of brain functions corresponding to subjective perception or cognition. In addition, dynamic rating‐based decoding is not restricted to fMRI and can be applied to other psychological and neural measurement devices to enhance our understanding of the association between psychological signals and dynamic perceptions. Future research could investigate the dynamic decoding of subjective experiences with electroencephalography (EEG), magnetoencephalography (MEG), functional near‐infrared spectroscopy (fNIRS), or other modalities that can be combined with sliding window analyses to close the gap between experimental and real‐world settings.

Since the decoding of dynamic perceptions has the potential to be used in a variety of areas, our experimental paradigm can also be applied to real‐world settings to decode cognitive dimensions and assess interactions based on psychological data (e.g., eye‐tracking, heart rate measurements). For example, likability can be predicted based on the dynamic decoding of psychological features in virtual reality, and interactions can change based on individual preferences for characters. Additionally, the decoding of dynamic cognitive dimensions can be used to reduce negative effects, such as visual fatigue and motion sickness, of interactive 3D content. Motion sickness, for instance, can be predicted from psychological data and potentially alleviated by reducing the field of view (Fernandes & Feiner, 2016) or constraining camera movement (Hu et al., 2019). Likewise, visual fatigue may be alleviated by reducing brightness (Benedetto et al., 2014).

Overall, the results of the present study show that dynamic subjective experiences can be decoded using naturalistic stimuli and suggest an alternative method to post‐task questionnaires for assessing subjective perception or cognition. We expect that our sliding window‐based approach will provide inspiration for future intelligent devices that can dynamically predict human perception or cognition from brain or psychological signals to maximize usability, comfort, and safety.

## AUTHOR CONTRIBUTIONS

Conceptualization, data curation, formal analysis, investigation, methodology, project administration, visualization, writing – original draft: Uijong Ju. Conceptualization, methodology, writing – review & editing: Christian Wallraven.

## CONFLICT OF INTEREST STATEMENT

The authors declare no conflicts of interest.

## Supporting information


**Data S1.** Supporting information.

## Data Availability

The data that support the findings of this study are available on request from the corresponding author. The data are not publicly available due to privacy or ethical restrictions.
