# Supplementary material for: Decoding the dynamic perception of risk and speed using naturalistic stimuli: A multivariate, whole‐brain analysis
Source: Hum Brain Mapp. 2024 Mar 15;45(4):e26652. doi: 10.1002/hbm.26652 (PMC10941534; doi:10.1002/hbm.26652)
Supplement: Supplementary file 1 — Data S1. Supporting information. [file HBM-45-e26652-s001.docx]

**Supporting methods and results**

**Evaluation study**

A total of 78 right-handed participants were recruited for the evaluation experiment (44 males, mean age=23.5 years (SD=3.27)). All participants were university students and had been in possession of a driving license for more than 1 year (mean=3.7 years, SD=2.21). Participants reported no problems watching the videos and no psychiatric or neurological disorders. A 27-inch monitor (LCD monitor; Samsung; Suwon, South Korea) was used to display the stimulus, and a joystick (Action Joystick; Seoul, South Korea) interface was used to enable continuous ratings of subjective risk and speed using the right hand.

Before the experiment, participants were informed about the aim of the study, were instructed to dynamically rate subjective risk during the first experimental block and subjective speed during the second block while watching seven 2 minute-long randomly presented videos, and provided informed consent. In addition, we explained to them that when the first block started, a 30-second baseline screen would be presented to inform them of the current aim (rate driving risk), and that the driving videos would then be displayed with a sliding bar that they had to use to rate their perceived risk. After the first block, the 30-second baseline screen was again presented to inform participants of the current task (rate driving speed) and instruct them to follow the same procedure. Dynamic ratings were recorded for each frame and averaged across all ratings to select the four most suiTABLE videos for the fMRI study.

Next, we conducted a repeated-measures ANOVA to compare risk and speed perception across the seven videos; the analysis yielded significant differences for both measures (risk: F(6)=13.086, p<.001; speed: F(6)=31.961, p<.001). We then conducted a post-hoc analysis to identify the videos that contained high and low risk situations as well as passages driven at high and low speed, and found that Video 1 (Video 3: t(77)=5.49, p<.001; Video 4: t(77)=7.70, p<.001; Video5: t(77)=6.67, p<.001; Video 6: t(77)=10.27, p<.001; Video 7: t(77)=7.63, p<.001) and Video 2 (Video 3: t(77)=5.08, p<.001; Video 4: t(77)=7.08, p<.001; Video 5: t(77)=6.28, p<.001; Video 6: t(77)=10.16, p<.001; Video 7: t(77)=7.72, p<.001) were recorded at significantly higher speeds than the other videos. We then ran an additional post-hoc analysis on perceived risk to identify the videos that contained high- and low-risk passages to pair them with Videos 1 and 2. Only Video 4 showed no significant differences in risk compared to Video 2 (t(77)=0.16, p=.87), while Video 3 showed a significantly higher risk compared to Video 2 and Video 4 (Video 2:t(77)=4.78, p<.001; Video 4:t(77)=4.86, p< .001) and no significantly different risk compared to Video 1 (t(77)=0.57, p=.573) (see FIGURE S1 and TABLE S1). We selected Videos 1, 2, 3, and 4 to represent the maximum range of high and low risk as well as high and low speed. Based on these results, we chose four of the seven videos for the fMRI experiment (see TABLE S2 for detailed post-hoc analysis results for all seven videos).

**Behavioral data analysis**

**Correlation between risk and speed measures**

We conducted a correlational analysis to investigate correlated between speed and risk measures that include average dynamic rating and post-task behavioral rating. Results showed that all measures were significantly correlated each other and post-rating risk showed highest correlations with average dynamic rating (r = 0.76, p < 0.001) and post-rating speed showed highest correlations with average dynamic speed (see Fig S2). Results showed that high speed positive correlated with high risk whereas their correlations were relatively smaller than correlation between same behavioral ratings.

Differences between men and women and influences of driving experiences.

We conducted an independent sample t-tests to investigate differences between men and women participants for behavioral ratings. Results showed that across all videos, there were no significant differences and only dynamic risk rating in video 1 showed trends that female participants rate higher perceived risk than male participants (t(29) = 1.90, p = 0.068) which showed that perceived risk were not significantly influenced by sex. Next, we conducted a correlational analysis between driving experiences and found that only post-task rating, video 4 showed significant correlations with driving experience (r = -0.39, p =0.032) and all other showed no significant correlations. Overall, in the present study, influences of sex or driving experiences on perceived risk and speed were relatively small.

**TABLE S1** Behavioral ratings for all seven videos.

|  | Video 1 | Video 2 | Video 3 | Video 4 | Video 5 | Video 6 | Video 7 |
| --- | --- | --- | --- | --- | --- | --- | --- |
| Dynamic average risk (SD) | 3.16 (0.65) | 2.82 (0.72) | 3.20 (0.54) | 2.81 (0.63) | 3.05 (0.63) | 3.10 (0.55) | 2.62 (0.64) |
| Dynamic average speed (SD) | 3.55 (0.43) | 3.54 (0.48) | 3.22 (0.54) | 3.13 (0.48) | 3.16 (0.50) | 2.87 (0.45) | 3.05 (0.49) |

SD, standard deviation


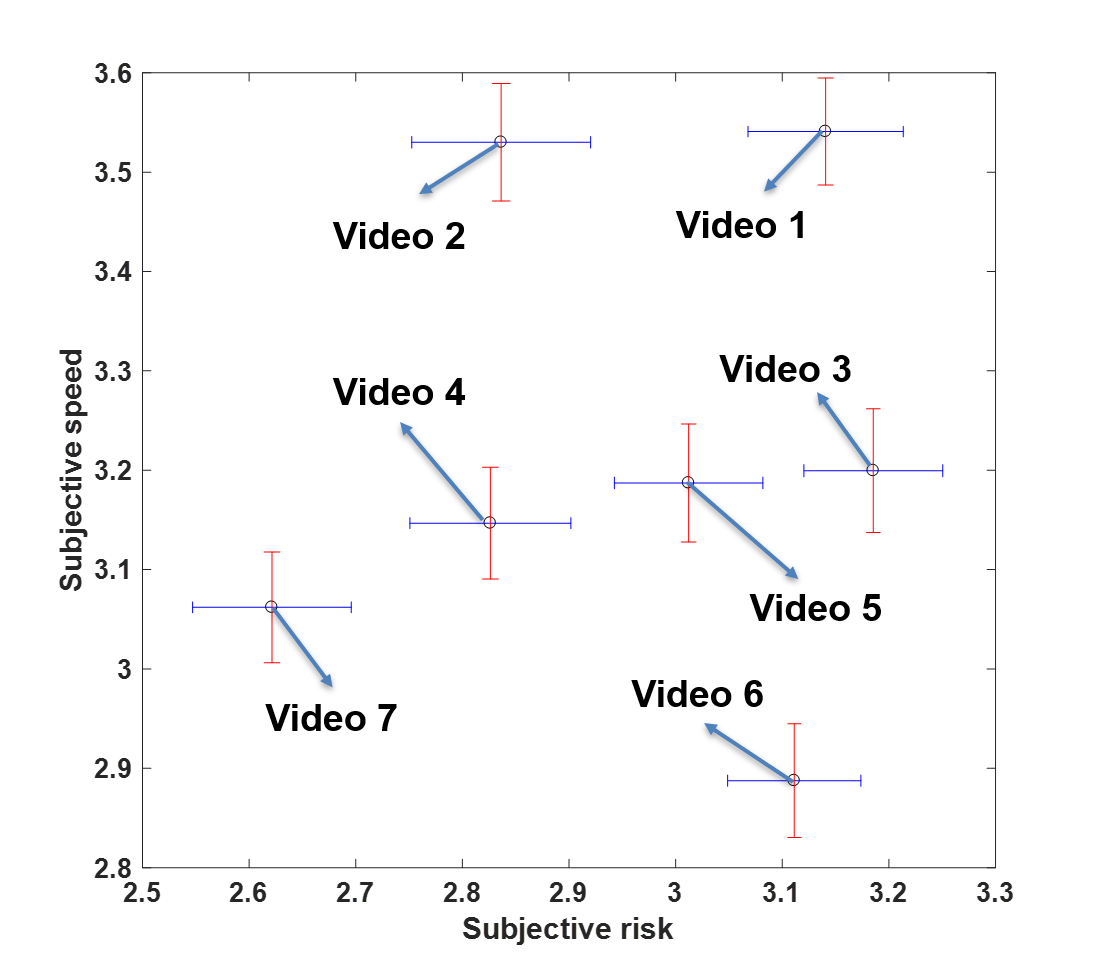


**FIGURE S1** 2D scatter plot with errorbars for subjective risk and speed in the behavioral experiment conducted to select videos for the fMRI experiment. Video 1, 2, 3, and 4 were selected to represent high and low risk as well as high and low speed.

**TABLE S2** Post-hoc analysis of risk and speed ratings derived from the evaluation experiment.

| Risk | Video 1 | Video 2 | Video 3 | Video 4 | Video 5 | Video 6 | Video 7 |
| --- | --- | --- | --- | --- | --- | --- | --- |
| Video 1 | 1 | **<.001^**^** | 0.573 | **.001^*^** | .182 | .477 | **<.001^**^** |
| Video 2 | **<.001^**^** | 1 | **<.001^**^** | 0.87 | .014 | **.002^*^** | .03 |
| Video 3 | .573 | **<.001^**^** | 1 | **.001^*^** | .082 | .194 | **<.001^**^** |
| Video 4 | **<.001^**^** | .87 | **<.001^**^** | 1 | .005 | **<.001** | .02 |
| Video 5 | .182 | .014 | .082 | .005 | 1 | .525 | **<.001^**^** |
| Video 6 | .477 | **.002^*^** | .194 | **.001^*^** | .525 | 1 | **<.001^**^** |
| Video 7 | **<.001^**^** | .030 | .001 | .02 | **<.001^**^** | **<.001^**^** | 1 |
| Speed | Video 1 | Video 2 | Video 3 | Video 4 | Video 5 | Video 6 | Video 7 |
| Video 1 | 1 | .857 | **<.001^**^** | **<.001^**^** | **<.001^**^** | **<.001^**^** | **<.001^**^** |
| Video 2 | .857 | 1 | **<.001^**^** | **<.001^**^** | **<.001^**^** | **<.001^**^** | **<.001^**^** |
| Video 3 | **<.001^**^** | **<.001^**^** | 1 | .193 | .139 | **<.001^**^** | .023 |
| Video 4 | **<.001^**^** | **<.001^**^** | .193 | 1 | .733 | **<.001^**^** | .188 |
| Video 5 | **<.001^**^** | **<.001^**^** | .139 | .733 | 1 | **<.001^**^** | .127 |
| Video 6 | **<.001^**^** | **<.001^**^** | **<.001^**^** | **<.001^**^** | **<.001^**^** | 1 | **.001^*^** |
| Video 7 | **<.001^**^** | **<.001^**^** | .023 | .188 | .127 | **.001^*^** | 1 |

***p<.005, **p<.001**

**TABLE S3**. Post-hoc analysis of risk and speed ratings recorded after the experiment.

| Stimulus 1 | Stimulus 2 | Mean risk differences (Stimulus 1- Stimulus 2) | Sig. | 95% confidence interval for Difference | |
| --- | --- | --- | --- | --- | --- |
|  |  |  |  | Lower bound | Upper bound |
| Video 1 | Video 2 | 0.216 | .058 | -0.007 | 0.439 |
|  | Video 3 | 0.141 | .335 | -0.153 | 0.436 |
|  | Video 4 | 0.517 | **<.001** | 0.262 | 0.771 |
| Video 2 | Video 3 | -0.074 | .518 | -0.306 | 0.158 |
|  | Video 4 | 0.301 | **.010** | 0.077 | 0.524 |
| Video 3 | Video 4 | 0.375 | **.005** | 0.124 | 0.630 |
| Stimulus 1 | Stimulus 2 | Mean speed differences (Stimulus 1- Stimulus 2) | Sig. | 95% confidence interval for Difference | |
|  |  |  |  | Lower bound | Upper bound |
| Video 1 | Video 2 | -0.195 | .160 | -0.471 | 0.081 |
|  | Video 3 | 0.356 | **.004** | 0.125 | 0.587 |
|  | Video 4 | 0.527 | **<.001** | 0.285 | 0.769 |
| Video 2 | Video 3 | 0.551 | **<.001** | 0.265 | 0.836 |
|  | Video 4 | 0.721 | **<.001** | 0.360 | 1.075 |
| Video 3 | Video 4 | 0.171 | .278 | -0.145 | 0.487 |

See TABLE 1 for descriptive statistics of the results.

**TABLE S4.** Maximum and minimum ratings as well as dynamic rating changes across all participants for the evaluation and the fMRI experiment.

|  | Video 1 | Video 2 | Video 3 | Video 4 |
| --- | --- | --- | --- | --- |
| Actual maximum speed | 272 km/h | 218 km/h | 242 km/h | 171 km/h |
| Actual minimum speed | 73 km/h | 67 km/h | 78 km/h | 56 km/h |
| Evaluation experiment maximum risk rating (SD) | 4.23 (0.66) | 3.95 (0.69) | 4.33 (0.60) | 4.00 (0.67) |
| Evaluation experiment minimum risk rating (SD) | 2.00 (0.69) | 1.72 (0.71) | 1.95 (0.65) | 1.69 (0.69) |
| Evaluation experiment maximum speed rating (SD) | 4.44 (0.52) | 4.51 (0.52) | 4.21 (0.61) | 4.17 (0.61) |
| Evaluation experiment minimum speed rating (SD) | 2.00 (0.69) | 1.72 (0.71) | 1.95 (0.65) | 1.69 (0.69) |
| fMRI experiment maximum risk rating (SD) | 4.05 (0.52) | 3.96 (0.56) | 3.99 (0.52) | 3.94 (0.55) |
| fMRI experiment minimum risk rating (SD) | 1.74 (0.67) | 1.90 (0.76) | 2.03 (0.56) | 1.94 (0.61) |
| fMRI experiment maximum speed rating (SD) | 4.08 (0.50) | 4.27 (0.52) | 4.11 (0.43) | 4.09 (0.50) |
| fMRI experiment minimum speed rating (SD) | 2.25 (0.66) | 2.29 (0.59) | 2.21 (0.57) | 2.11 (0.59) |
| Evaluation experiment number of rating changes in risk perception (SD) | 31.95 (11.73) | 31.13 (11.10) | 32.64 (11.67) | 31.19 (12.12) |
| Evaluation experiment number of rating changes in speed perception (SD) | 30.22 (11.00) | 30.60 (12.07) | 29,44 (11.49) | 29.60 (11.71) |
| fMRI experiment number of rating changes in risk perception (SD) | 34.65 (11.47) | 33.23 (11.52) | 33.90 (9.95) | 33.87 (11.29) |
| fMRI experiment number of rating changes in speed perception (SD) | 30.10 (11.77) | 29.81 (10.17) | 29,19 (11.07) | 30.52 (10.02) |

SD, standard deviation

|  | Video 1 | Video 2 | Video 3 | Video 4 |
| --- | --- | --- | --- | --- |
| Post-task risk rating_men (std) | 3.21 (0.77) | 2.97 (0.68) | 3.11 (0.85) | 2.66 (0.76) |
| Post-task risk rating_women (std) | 3.32 (0.62) | 3.16 (0.56) | 3.10 (0.77) | 2.88 (0.87) |
| Average dynamic risk_rating men (std) | 2.89 (0.42) | 2.94 (0.40) | 3.08 (0.40) | 2.90 (0.40) |
| Average dynamic risk_rating women (std) | 3.18 (0.34) | 3.13 (0.45) | 3.12 (0.49) | 2.87 (0.40) |
| Post-task speed rating_men (std) | 3.52 (0.65) | 3.60 (0.74) | 3.25 (0.72) | 2.89 (0.81) |
| Post-task speed rating_women (std) | 3.42 (0.60) | 3.85 (0.52) | 2.88 (0.54) | 3.11 (0.81) |
| Average dynamic speed_rating men (std) | 3.10 (0.43) | 3.26 (0.39) | 3.16 (0.43) | 3.19 (0.40) |
| Average dynamic speed_rating (std)women | 3.19 (0.44) | 3.27 (0.21) | 3.05 (0.42) | 2.93 (0.47) |

**TABLE S5.** . Post-task rating and average dynamic ratings for men and women participants.


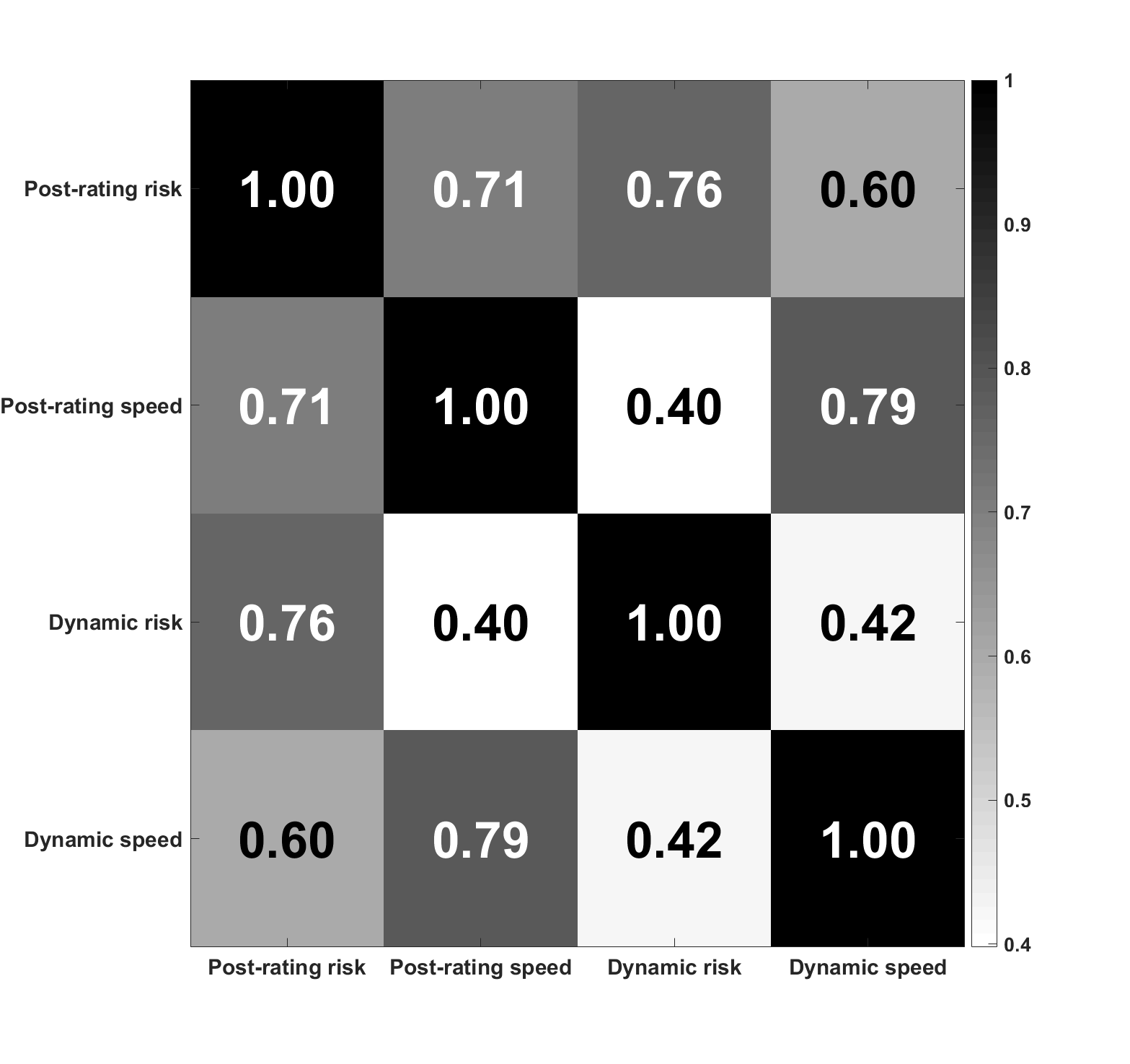


**FIGURE S2.** Correlation matrix for the post-task risk, speed rating and averaged dynamic risk and speed.


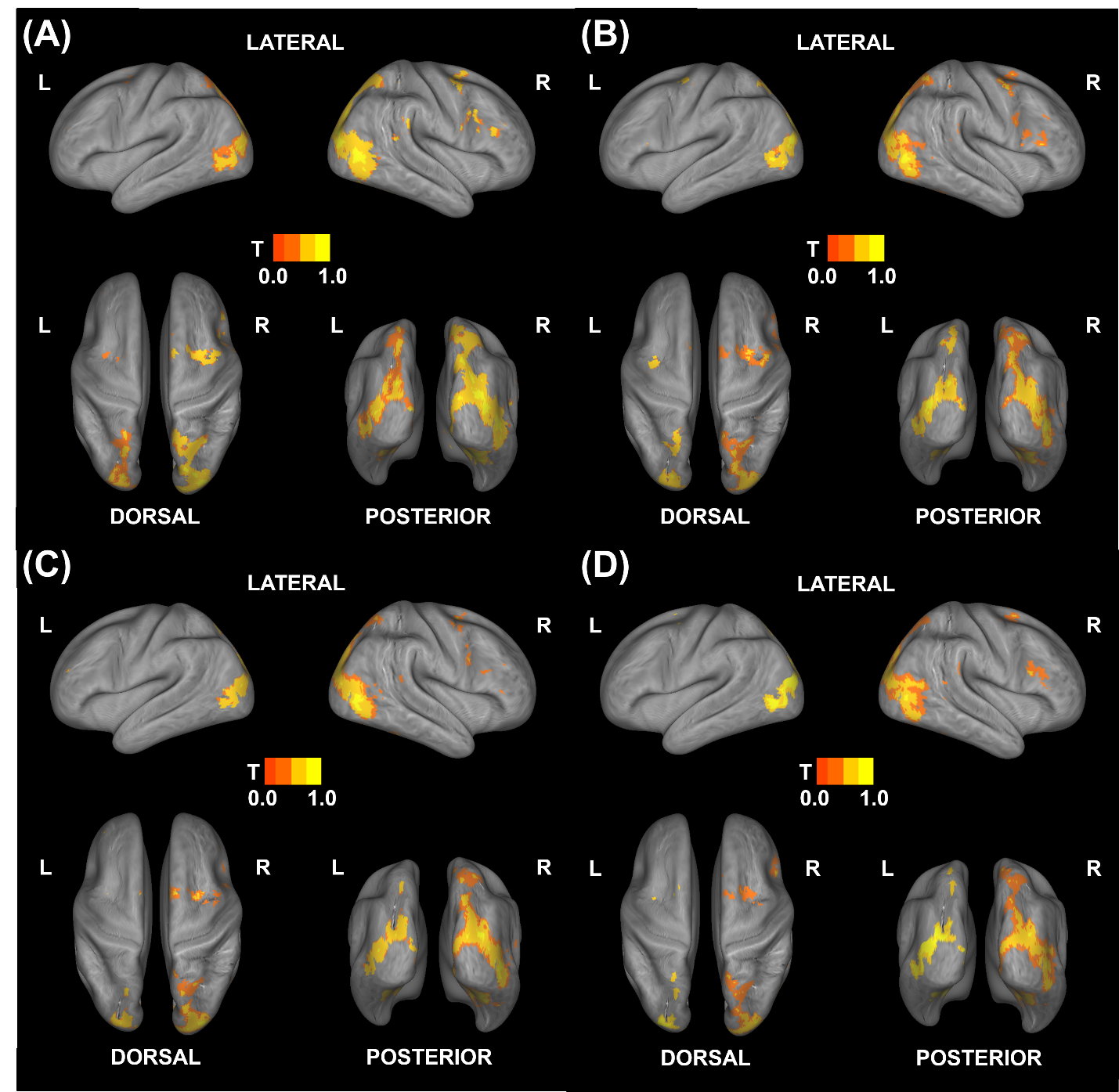


**FIGURE S3.** Univariate analysis (general linear model) for task versus baseline in risk rating condition (p <.05 FWE corrected). (A) video 1 (B) video 2 (c) video 3 (d) video 4. All t-values are rescaled to 0-1 for better comparison.


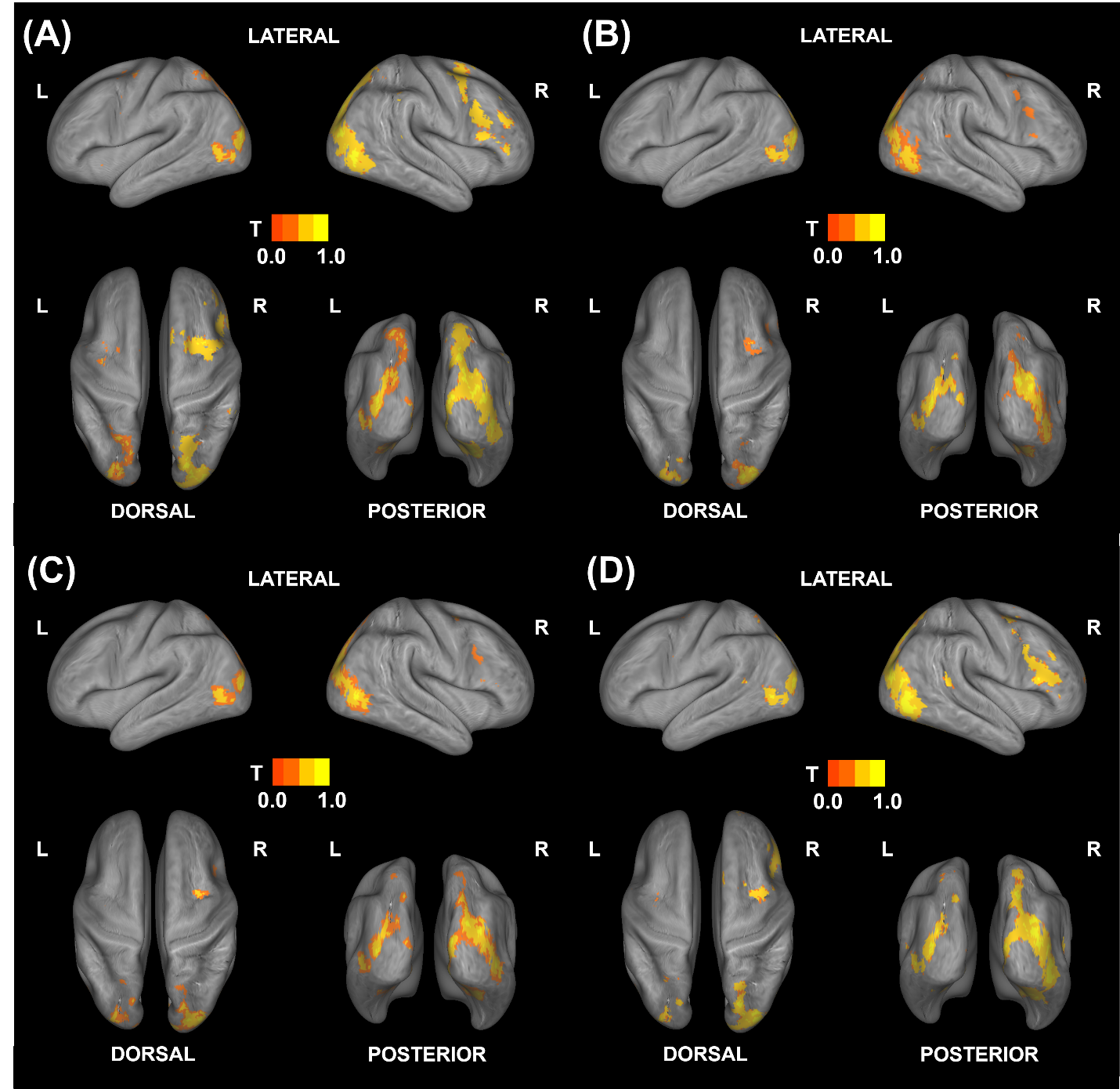


**FIGURE S4.** Univariate analysis (general linear model) for task versus baseline in speed rating condition (p <.05 FWE corrected). (A) video 1 (B) video 2 (c) video 3 (d) video 4. All t-values are rescaled to 0-1 for better comparison.


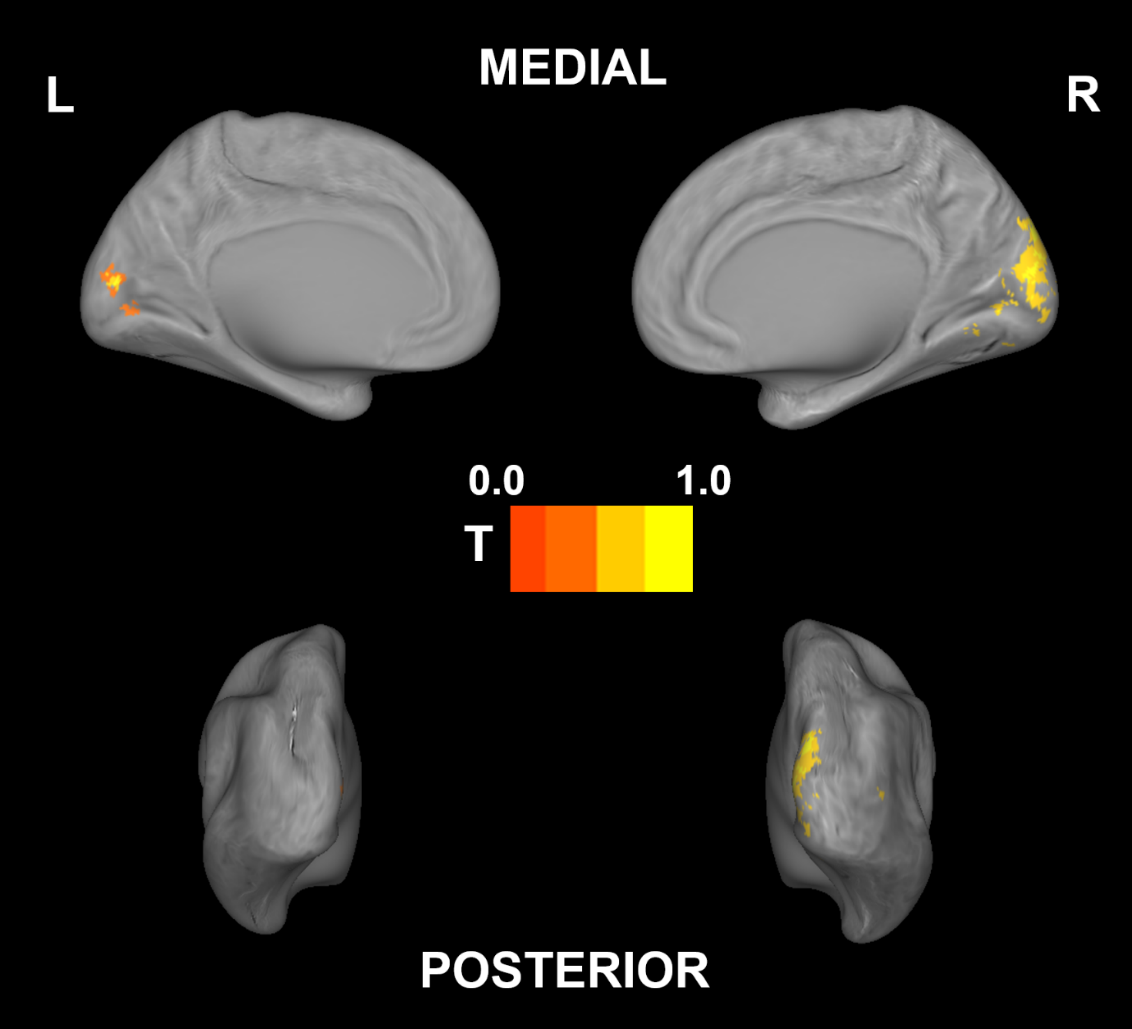


**FIGURE S5.** Correlational searchlight analysis for brain regions that can decode averaged dynamic risk for each video. Results are p<.001 uncorrected for visualization. All t-values are rescaled to 0-1 for better comparison.

**TABLE S6.** Location information of the correlational searchlight analysis.

| Region (AAL) | Peak Voxel | Z-score | Number of voxels |
| --- | --- | --- | --- |
| Cuneus | -2 -82 8 | 6.78 | 494 |
| Lingual gyrus | 12 – 68 -2 | 4.07 | 31 |
| Middle Occipital gyrus | 24 -88 2 | 4.40 | 29 |
| Middle Temporal gyrus | 42 -74 12 | 4.02 | 11 |
| Posterior Cingulate | 6 -72 10 | 3.77 | 20 |
| Subgyrus | -18 -12 58 | 3.92 | 21 |

All analyses are p<.05 FDR-corrected. See FIGURE S2 for a visualization of the results.


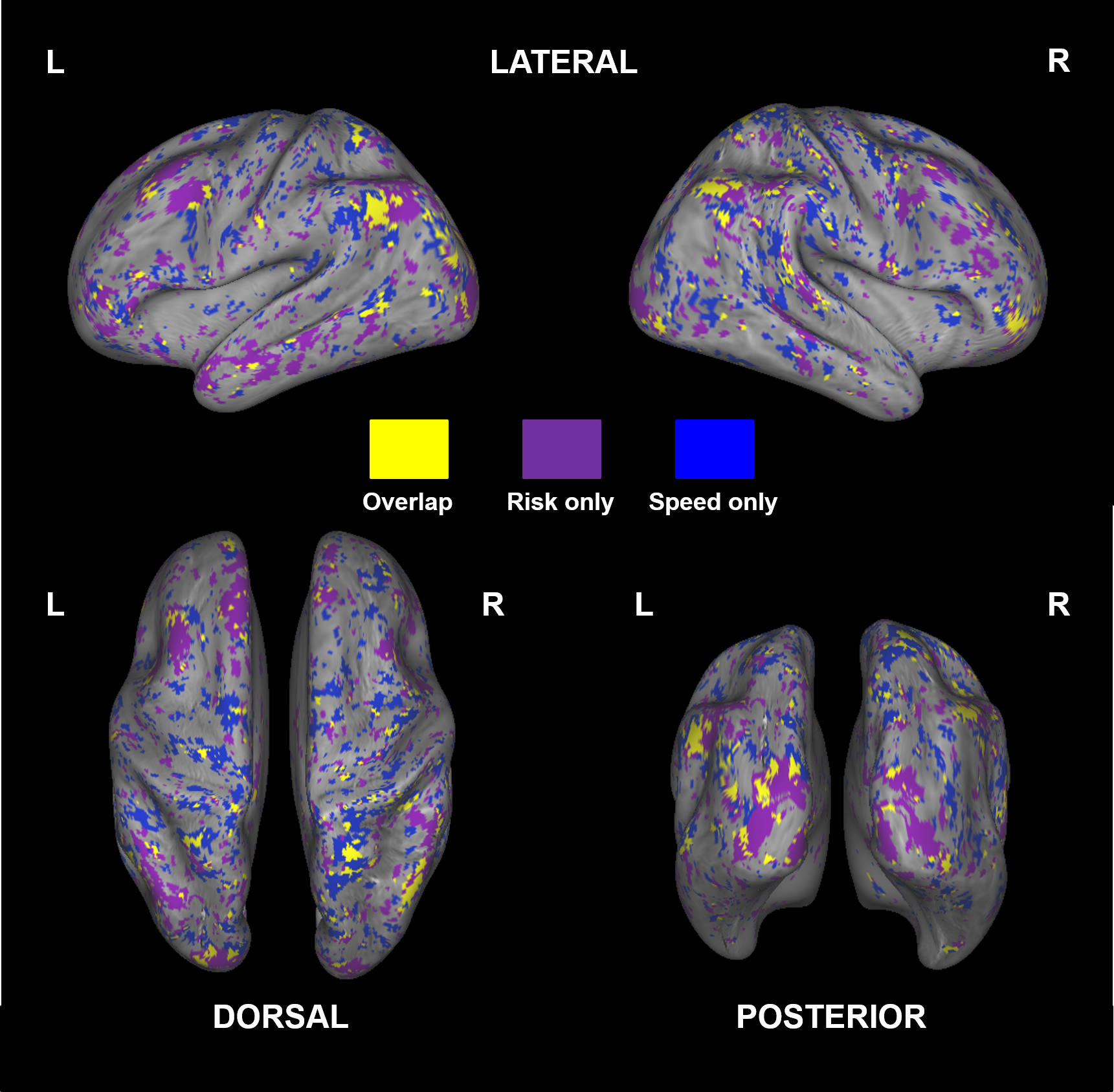


**FIGURE S6.** Sliding window-based correlation analysis for overlapped risk and speed perception (p<.05 FDR-corrected). (A) Risk perception. (B) Speed perception. Values are rescaled to 0–1 to display frequency of significance from 1–30 scans to 31–60 scans.

**TABLE S7.** Location information of dynamic rating-based correlation analysis for overlapped, only risk, and only speed regions.

|  | Region (AAL) | Number of voxels |
| --- | --- | --- |
| Overlap | Cuneus | 198 |
|  | Inferior frontal gyrus | 144 |
|  | Inferior parietal lobule | 270 |
|  | Medial frontal gyrus | 131 |
|  | Middle frontal gyrus | 382 |
|  | Middle occipital gyrus | 186 |
|  | Middle temporal gyrus | 253 |
|  | Postcentral gyrus | 232 |
|  | Precentral gyrus | 150 |
|  | Precuneus | 141 |
|  | Superior frontal gyrus | 263 |
|  | Superior temporal gyrus | 107 |
|  | Supramarginal gyrus | 125 |
| Only risk | Cingulate gyrus | 513 |
|  | Cuneus | 1145 |
|  | Inferior frontal gyrus | 963 |
|  | Inferior parietal lobule | 945 |
|  | Lingual gyrus | 597 |
|  | Medial frontal gyrus | 735 |
|  | Middle frontal gyrus | 1802 |
|  | Middle occipital gyrus | 795 |
|  | Middle temporal gyrus | 1224 |
|  | Postcentral gyrus | 528 |
|  | Precentral gyrus | 557 |
|  | Precuneus | 709 |
|  | Superior frontal gyrus | 1544 |
|  | Superior temporal gyrus | 793 |
| Only speed | Inferior frontal gyrus | 663 |
|  | Inferior parietal lobule | 708 |
|  | Medial frontal gyrus | 818 |
|  | Middle frontal gyrus | 1394 |
|  | Middle temporal gyrus | 917 |
|  | Postcentral gyrus | 1346 |
|  | Precentral gyrus | 1107 |
|  | Precuneus | 898 |
|  | Superior frontal gyrus | 992 |
|  | Superior temporal gyrus | 791 |

All analyses are p<.05 FDR-corrected, and reported clusters have more than 100 significant voxels for overlapped and 500 significant voxels for risk and speed regions. See FIGURE S3 for a visualization of the results.


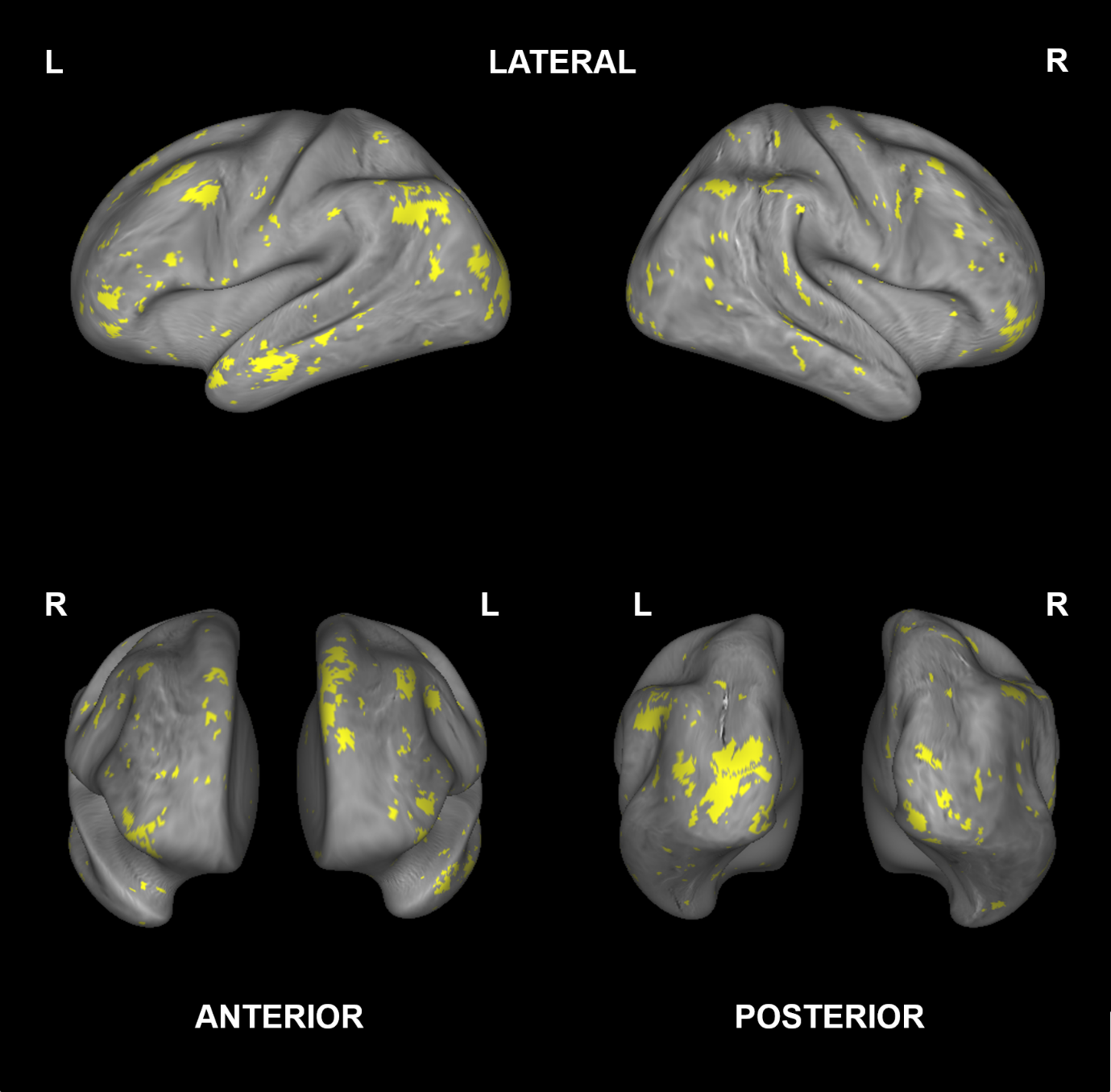


**FIGURE S7.** Correlation between beta estimates and dynamic behavioral ratings for risk perception using whole scans. Results are p<.05 FDR-corrected; common voxels across all four driving videos are indicated.


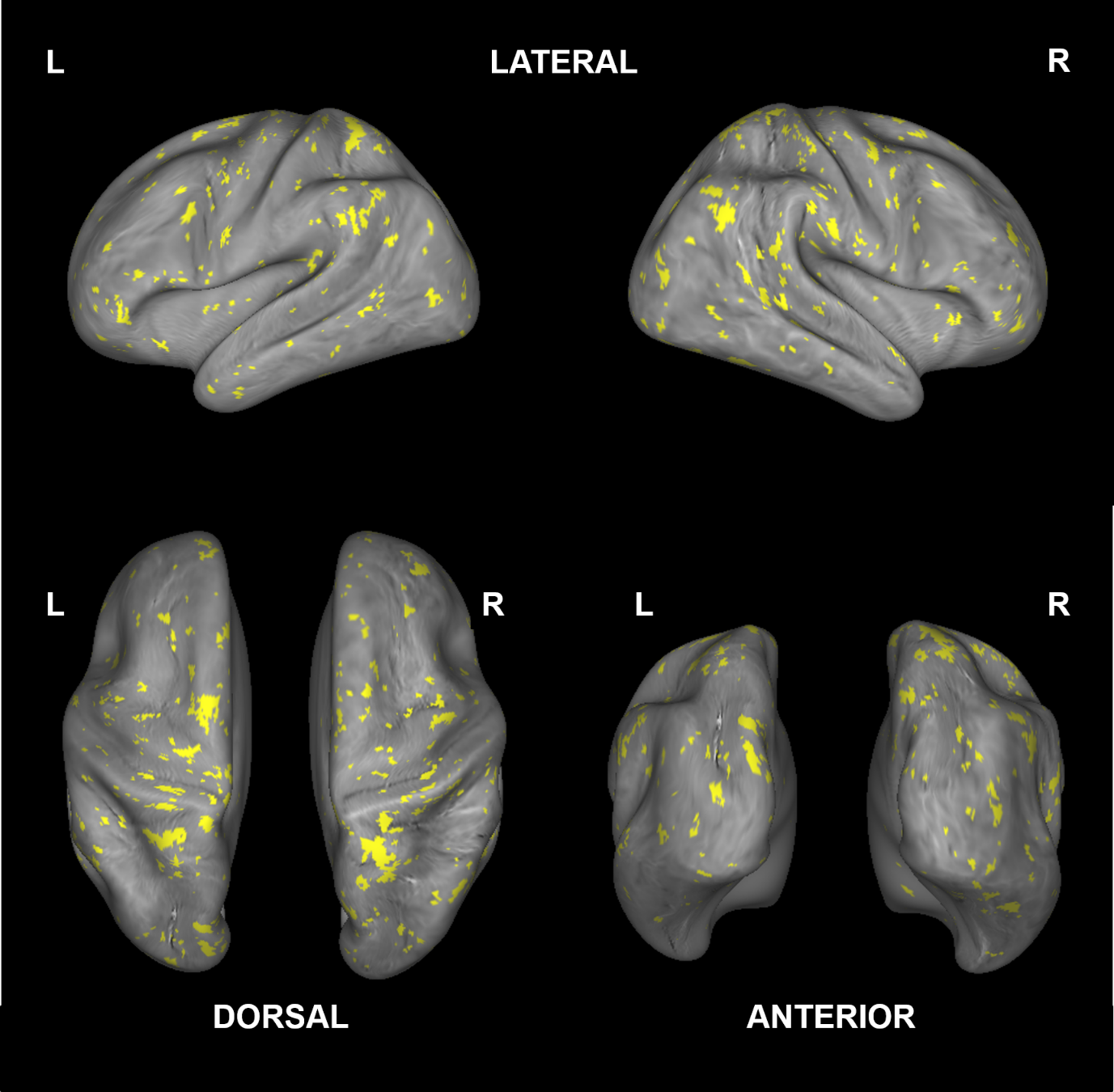


**FIGURE S8**. Correlation between beta estimates and dynamic behavioral ratings for speed perception using whole scans. Results are p<.05 FDR-corrected; common voxels across all four driving videos are indicated.

**TABLE S8.** Location information of the dynamic rating-based correlation analysis.

| Dynamic risk | Region (AAL) | Number of voxels | Dynamic speed | Region (AAL) | Number of voxels |
| --- | --- | --- | --- | --- | --- |
|  | Angular gyrus | 157 |  | Cingulate gyrus | 126 |
|  | Cingulate gyrus | 139 |  | Cuneus | 163 |
|  | Cuneus | 544 |  | Inferior frontal gyrus | 318 |
|  | Declive | 130 |  | Inferior parietal lobule | 361 |
|  | Inferior frontal gyrus | 326 |  | Lingual gyrus | 153 |
|  | Inferior parietal lobule | 359 |  | Medial frontal gyrus | 358 |
|  | Lingual gyrus | 180 |  | Middle frontal gyrus | 649 |
|  | Medial frontal gyrus | 223 |  | Middle occipital gyrus | 171 |
|  | Middle frontal gyrus | 764 |  | Middle temporal gyrus | 414 |
|  | Middle occipital gyrus | 402 |  | Paracentral lobule | 137 |
|  | Middle temporal gyrus | 612 |  | Postcentral gyrus | 655 |
|  | Parahippocampal gyrus | 126 |  | Precentral gyrus | 461 |
|  | Postcentral gyrus | 135 |  | Precuneus | 319 |
|  | Precentral gyrus | 177 |  | Superior frontal gyrus | 503 |
|  | Precuneus | 159 |  | Superior parietal lobule | 155 |
|  | Superior frontal gyrus | 720 |  | Superior temporal gyrus | 367 |
|  | Superior temporal gyrus | 244 |  | Supramarginal gyrus | 168 |

All analyses are p<.05 FDR-corrected, and reported clusters have more than 100 voxels. See FIGURE S4 for a visualization of the risk results and FIGURE S5 for a visualization of the speed results.


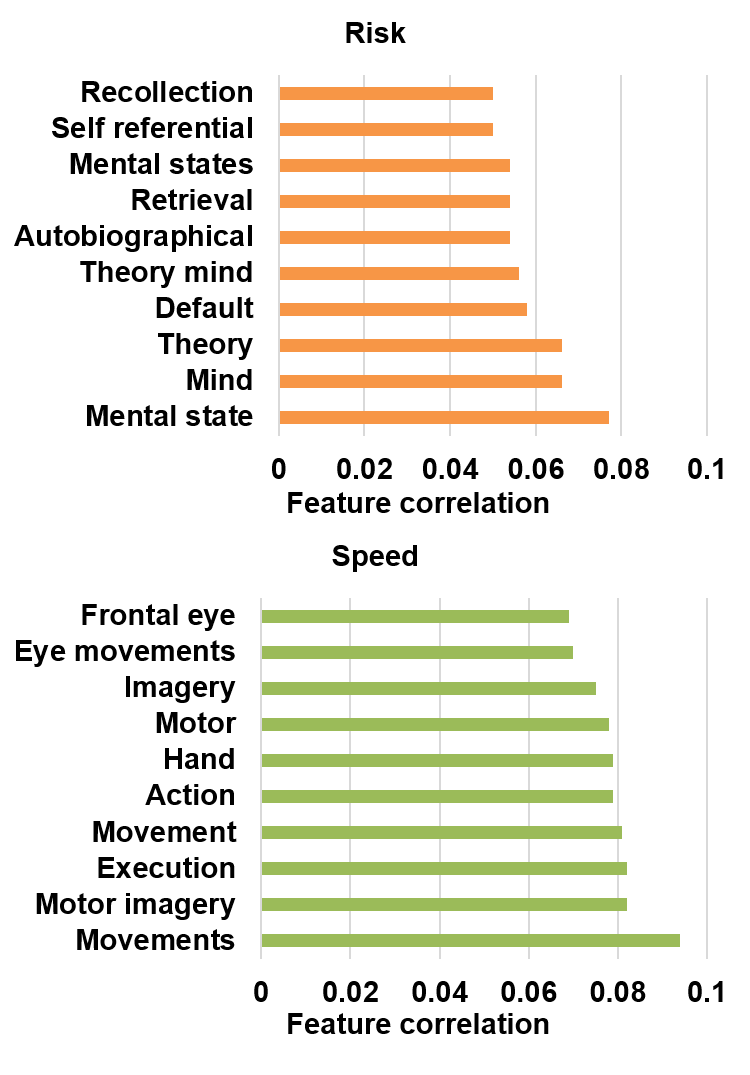


**FIGURE S9.** Psychological features associated with the activation map (see FIGURE S4-S5), extracted using the decoding function of Neurosynth.


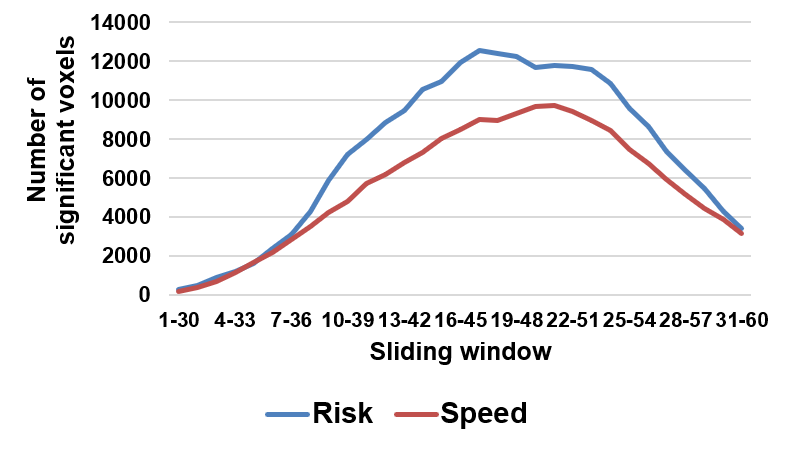


**FIGURE S10.** Number of significant voxel changes from the 1–30 to the 31–60 sliding window for dynamic risk and speed perception. Significant voxels represent common voxels activated across all four videos.


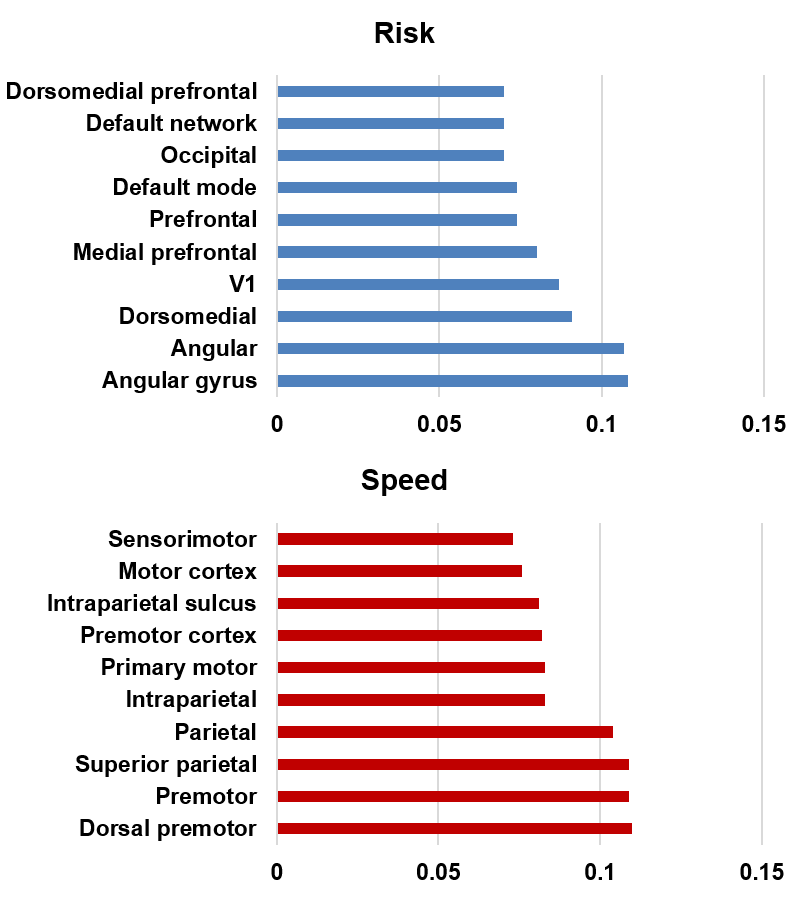


**FIGURE S11.** Anatomical features associated with the overall dynamic ratings in FIGURE 5, extracted using the decoding function of Neurosynth.

**
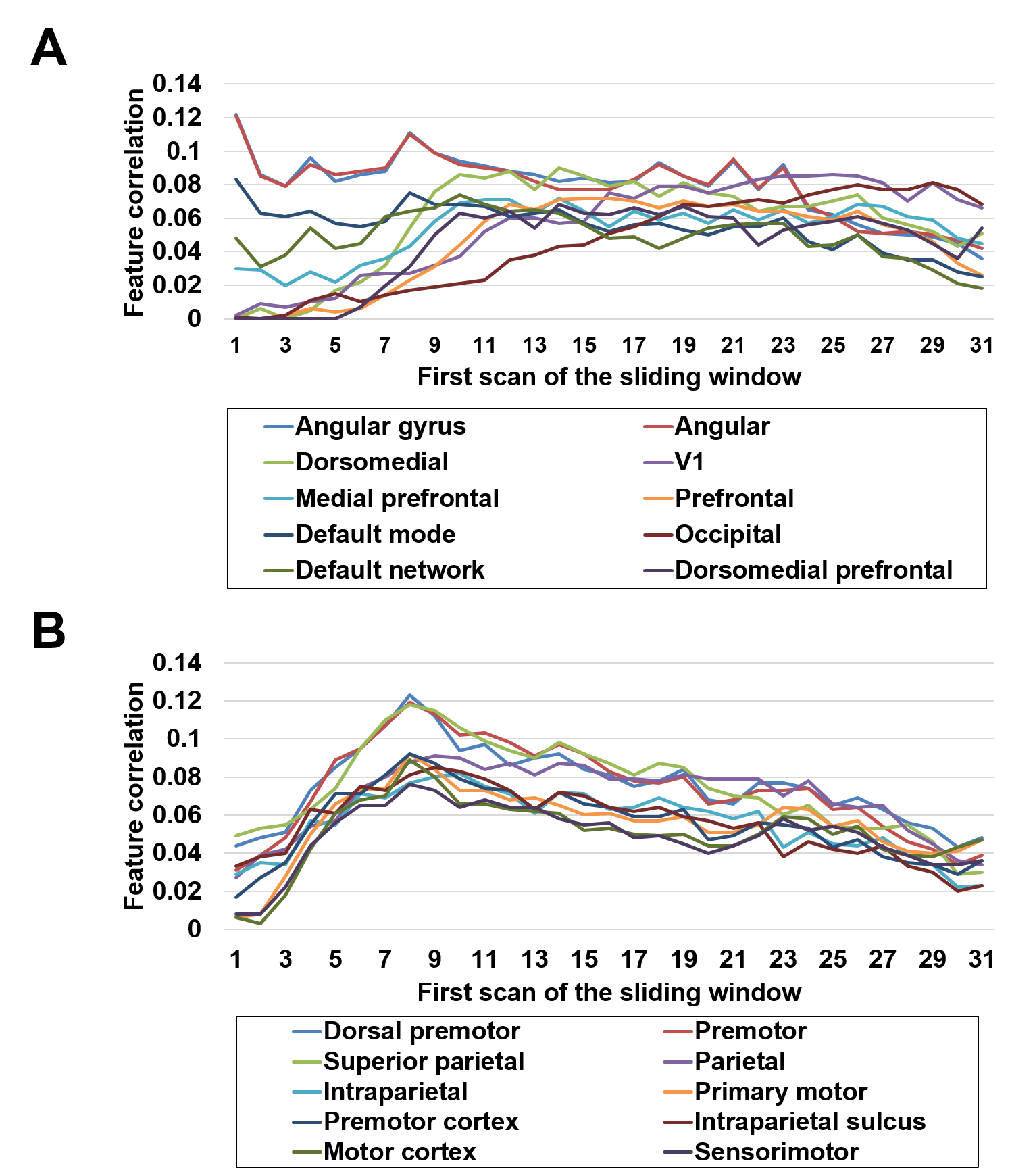
**

**FIGURE S12.** Anatomical features associated with the dynamic ratings from sliding window 1–30 to 31–60, extracted using the decoding function of Neurosynth. (A) Risk perception. (B) Speed perception.
